# Bacterial growth and environmental adaptation via thiamine biosynthesis and thiamine-mediated metabolic interactions

**DOI:** 10.1093/ismejo/wrae157

**Published:** 2024-08-12

**Authors:** Xihui Xu, Can Li, Weimiao Cao, Lulu Yan, Lulu Cao, Qi Han, Minling Gao, Yahua Chen, Zhenguo Shen, Jiandong Jiang, Chen Chen

**Affiliations:** College of Life Sciences, Nanjing Agricultural University, Nanjing 210095, China; College of Life Sciences, Nanjing Agricultural University, Nanjing 210095, China; College of Life Sciences, Nanjing Agricultural University, Nanjing 210095, China; College of Life Sciences, Nanjing Agricultural University, Nanjing 210095, China; College of Life Sciences, Nanjing Agricultural University, Nanjing 210095, China; College of Life Sciences, Nanjing Agricultural University, Nanjing 210095, China; Department of Materials and Environmental Engineering, Shantou University, Shantou 515063, China; College of Life Sciences, Nanjing Agricultural University, Nanjing 210095, China; Jiangsu Collaborative Innovation Center for Solid Organic Waste Resource Utilization, Nanjing Agricultural University, Nanjing 210095, China; College of Life Sciences, Nanjing Agricultural University, Nanjing 210095, China; Jiangsu Collaborative Innovation Center for Solid Organic Waste Resource Utilization, Nanjing Agricultural University, Nanjing 210095, China; College of Life Sciences, Nanjing Agricultural University, Nanjing 210095, China; College of Life Sciences, Nanjing Agricultural University, Nanjing 210095, China; Jiangsu Collaborative Innovation Center for Solid Organic Waste Resource Utilization, Nanjing Agricultural University, Nanjing 210095, China

**Keywords:** microbial community, bacterial evolution, thiamine, metabolic interactions, metabolic modeling, synthetic community

## Abstract

Understanding the ancestral transition from anaerobic to aerobic lifestyles is essential for comprehending life’s early evolution. However, the biological adaptations occurring during this crucial transition remain largely unexplored. Thiamine is an important cofactor involved in central carbon metabolism and aerobic respiration. Here, we explored the phylogenetic and global distribution of thiamine-auxotrophic and thiamine-prototrophic bacteria based on the thiamine biosynthetic pathway in 154 838 bacterial genomes. We observed strong coincidences of the origin of thiamine-synthetic bacteria with the “Great Oxygenation Event,” indicating that thiamine biosynthesis in bacteria emerged as an adaptation to aerobic respiration. Furthermore, we demonstrated that thiamine-mediated metabolic interactions are fundamental factors influencing the assembly and diversity of bacterial communities by a global survey across 4245 soil samples. Through our newly established stable isotope probing–metabolic modeling method, we uncovered the active utilization of thiamine-mediated metabolic interactions by bacterial communities in response to changing environments, thus revealing an environmental adaptation strategy employed by bacteria at the community level. Our study demonstrates the widespread thiamine-mediated metabolic interactions in bacterial communities and their crucial roles in setting the stage for an evolutionary transition from anaerobic to aerobic lifestyles and subsequent environmental adaptation. These findings provide new insights into early bacterial evolution and their subsequent growth and adaptations to environments.

## Introduction

Life on Earth originated and evolved in anoxic environments [1–3]. The “Great Oxygenation Event” (GOE) ~2.4 billion years ago (Bya) is recognized as the most geologically critical environmental change impacting the history of life [4, 5]. As oxygen levels rose on the early Earth during GOE, anaerobic life either began to utilize oxygen to adapt to aerobic environments or retreated to anoxic environments, resulting in various biochemical reactions of anaerobic and aerobic metabolism during this evolutionary process [6, 7]. For example, the electron transport chain is essential for energy provision of aerobic lifestyles [8], and their origin and evolution are crucial for setting the stage for an evolutionary transition from anaerobic to aerobic lifestyles. Although the significance of emergences of the aerobic respiratory chain and antioxidant systems has been widely acknowledged [9–11], the biological adaptations that occur during this pivotal transition remain largely unexplored.

Thiamine (also known as vitamin B1, VB1) and thiamine pyrophosphate (TPP, the biologically active form of thiamine) are essential cofactors for the activity of many key enzymes that participate in central carbon metabolism pathways, including glycolysis and the tricarboxylic acid (TCA) cycle [12–14]. For example, TPP is crucial in the pyruvate dehydrogenase complex (PDHC), which catalyzes the conversion of pyruvate to acetyl-CoA, and the integrated TPP moiety receives electrons in the redox processes conducted by the PDHC. Similar functions of TPP are found in the α-ketoglutarate dehydrogenase complex (KGDHC), which participates in the decarboxylation of α-ketoglutarate. These results show that TPP is a crucial molecule indirectly involved in the electron transport chain and essential for adequate aerobic metabolism [14]. Therefore, we speculated that the thiamine biosynthetic pathway originated and evolved in response to the rising oxygen caused by GOE and was involved in the transition to aerobicity.

Both thiamine-auxotrophic and thiamine-prototrophic bacteria have been widely reported, and thiamine-mediated metabolic interactions between them have been frequently detected [15–17]. Considering the potential evolution process of thiamine biosynthetic pathway and vital roles of thiamine in metabolism mentioned above, we hypothesized that thiamine-mediated metabolic interactions between thiamine-auxotrophic and thiamine-prototrophic bacteria could be globally ubiquitous, thereby serving as a fundamental factor influencing the assembly and diversity of microbial communities. In contrast, thiamine-mediated metabolic interactions within thiamine-prototrophic bacteria are significantly more challenging to detect, leading to their under-appreciation. The extent to which thiamine-mediated metabolic interactions within thiamine-prototrophic bacteria affect bacterial community functions remains unknown. One major obstacle is that tools to elucidate complex metabolic interactions within bacterial communities in natural environments are still lacking.

The function and activity of microbial communities largely depend on the complex metabolic interactions among strains in communities [18–20]. Thus, clarifying microbial metabolic interactions is an important precondition for better understanding the determining mechanism of microbial community functions and microbial community applications [21]. For example, synthetic communities (SynComs) have been proposed for applications of microbial communities [19, 22]. A key aspect of SynCom design involves manipulating community structure based on metabolic characteristics and interactions among microbial strains, thereby harnessing the combined metabolic capacities for constructing complex functions. Thus, exploring metabolic interactions within natural microbial communities is essential for designing SynComs [23]. Unfortunately, current studies of microbial metabolic interactions are often limited to simple microbial communities in the laboratory, and metabolic interactions under natural conditions are largely ignored.

Recently, metabolic modeling has attracted extensive attention as a computational tool for simulating the metabolic activities of microbial communities [24, 25]. Advances in simulation algorithms and computational tools have enabled the analysis of interspecific interactions in bacterial communities [19, 26]. The genome-scale metabolic model (GSMM) is a mathematical framework that can facilitate the prediction of multiscale phenotypes by optimizing the objective function of interest [26, 27]. Through the GSMM, models of different microbial communities can be constructed, and their metabolic characteristics can be simulated and compared. DNA stable isotope probing (SIP) technology has been successfully applied in identifying strains involved in specific functions [28, 29]. Unfortunately, SIP technology cannot further reveal the metabolic interactions between functional strains. Here, we made a first attempt to use a combined strategy of SIP and metabolic modeling to explore key metabolic interactions in a complex natural microbiome. We showed that the SIP-metabolic modeling strategy can not only identify functional strains but also analyze metabolic interactions between strains, compensating for shortcomings when used alone.

In recent decades, persistent organic pollutants (POPs) have emerged as a significant environmental concern [30]. Bioremediation is recognized as an economical, eco-friendly, and sustainable technology for degrading POPs in environments [31]. However, the survival and activity of exogenous inoculations are often hindered by the indigenous microbial communities and local habitats [32, 33]. SynComs, which harness interactions among exogenously introduced degrading strains and indigenous microbial strains, offer a promising approach to overcoming these limitations [19, 23]. Bromoxynil octanoate (BO) is a halogenated aromatic herbicide, with bromoxynil being its metabolic intermediate and active ingredient. BO and bromoxynil have been widely used for postemergence control of annual broadleaved weeds [34], yet their excessive and improper use has led to environmental pollution and ecosystem damage [25, 35]. BO-degrading bacteria are prevalent in soil, but they typically convert BO primarily into bromoxynil rather than completely mineralizing it [36–38]. Consequently, bromoxynil accumulates in soils as the predominant pollutant resulting from BO applications. Some bacteria, such as *Pseudoxanthomonas* sp. X-1 and *Comamonas* sp. 7D-2, cooperate metabolically to mineralize BO: the former degrades BO into bromoxynil, and the latter further degrades bromoxynil [23]. These functional strains are crucial for constructing SynComs with enhanced BO/bromoxynil-degrading efficiency.

In this study, we developed a method combining SIP and metabolic modeling to investigate metabolic interactions in soils and to construct SynComs aimed at enhancing BO/bromoxynil degradation. Our findings highlighted thiamine as a key compound mediating metabolic interactions within the SynCom during bromoxynil degradation. To assess the prevalence of thiamine-mediated metabolic interactions across bacterial communities and their impact on bacterial evolutionary processes, we explored the thiamine biosynthetic pathway in bacteria based on the available genomes (154 838 genomes) from NCBI and constructed time-calibrated bacterial phylogenetic trees to illuminate the evolutionary timescale of thiamine biosynthetic bacteria. We then conducted a global survey (4245 soils distributed across seven continents) of thiamine-auxotrophic and -prototrophic bacteria based on microbiome data generated by the Earth Microbiome Project (EMP). Our results promoted understanding of metabolic interactions in bacterial communities, highlighting thiamine-mediated metabolic interactions as an adaptive strategy for bacterial communities in response to environmental changes.

## Materials and methods

### Identification of thiamine-related genes

Both hidden Markov model (HMM)-based and BLASTP-based searches were performed to identify thiamine-related genes in selected genomes. The reference protein sequences (ThiC, ThiE, ThiG, and ThiL) were downloaded and artificially screened from UniProt and aligned using MAFFT v7.310 [39]. The resulting sequence alignment was visualized by Geneious Pro v4.8.3 and revised manually. HMM profiles were built on the curated alignments using HMMER v3.3.1 [40]. To collect homologs of thiamine-related genes, a total of 154 838 bacterial genomes were downloaded from NCBI. The HMM profile was searched against all 154 838 genomes using hmmsearch in HMMER. In parallel, using the reference protein sequences as the query, the BLASTP search was performed against all the above genomes using Diamond v2.0.2.140 [41]. The details for assessing the cutoffs of *E*-values and Hit scores are provide in Supplementary methods.

### Reconstruction of the bacterial species tree and molecular dating of the tree

To reconstruct the bacterial species tree, a total of 2435 genera were retrieved from the 154 838 bacterial genomes (2724 genera in total, and 289 genera with low-quality genomes were removed from the phylogenetic analysis). The completeness and contamination levels of these genomes were evaluated using CheckM2 [42]. The results indicated that the completeness of nearly all genomes (>96.6%) was above 99%, and contamination levels for nearly all genomes (>95.4%) were below 2% (Fig. S1). A representative genome was randomly selected for each genus to simplify the phylogenetic tree. Two archaeal genomes were used as outgroups. BUSCO genes have been widely used as markers for phylogenomic inference in diverse lineages [43]. Therefore, the bacteria_odb10 database containing 124 bacterial BUSCO single-copy orthologs was downloaded as the HMM profile, which was searched against all the representative genomes using HMMER. A total of 41 universal orthologs in bacterial genomes were obtained, and 20 single-copy orthologs were shared in the selected genomes of both bacteria and archaea, which were used as taxonomic marker genes for phylogenetic analysis. A concatenated alignment was generated for these 20 universally conserved orthologous proteins using MAFFT v7.310 [39] and trimmed by Gblocks v0.91b [44] using the default parameters to select conserved regions. The maximum-likelihood tree was built using IQ-tree v2.1.4-beta using the MFP mode with 1000 ultrafast bootstraps [45]. For molecular dating of the species tree, we reconstructed a phylogenetic tree with 148 genomes covering the main families in each phylum based on the above 20 single-copy orthologs. The divergence time of the species tree was estimated with MCMCTree from PAML v4.9i [46], which is a widely used MCMC-based tool for molecular dating. The detailed methods are proved in Supplementary methods.

### Global survey on the environmental distributions of thiamine-auxotrophic and thiamine-prototrophic bacteria

To avoid issues in combining multiple amplicons across diverse environments, the EMP used a unified standard workflow for sample collection, data production, and analysis, such as employing standard methods for DNA extraction, sequencing, and sequence preprocessing [47]. The microbial abundance table used in the study was derived from the Silva-based rarefied table generated by the EMP. A total of 4245 soil samples with geographic information were extracted from the raw dataset, which were collected across eight continents. Based on the results of identification of thiamine-related genes, we classified bacteria into four types: (i) “thiL-ECG,” strains harboring all four essential genes of *thiL*, *thiE*, *thiC*, and *thiG*; (ii) “thiL-others,” strains possessing *thiL* but lacking any of the genes *thiC*, *thiG*, or *thiE*; (iii) “NthiL-noneECG,” strains lacking all four genes; and (iv) “NthiL-others,” strains lacking *thiL* but possessing at least one of *thiC*, *thiG*, and *thiE*. To link genome-based thiamine assignments with 16s rRNA gene amplicon sequencing data from the EMP, we first assessed the percentage of these four types within each genus separately, using a dataset comprising 154 838 bacterial genomes. Our analysis revealed that most genera were dominated by a single type. Subsequently, we identified the overlap between two sets of bacteria at the genus level: those dominated by a single type based on genome data and those detected by 16S rRNA gene data from EMP, which were classified as “known” type for thiamine assignments in each soil sample. Due to incomplete coverage of bacterial taxa in NCBI genome datasets and the presence of unknown/unclassified taxa in 16S rRNA gene data, not all bacteria detected by 16S rRNA gene data had corresponding genomic data. Genera detected only by 16S rRNA gene data were classified as “unknown” type for thiamine assignments. Similar approaches have been used by previous studies [48–50]. One limitation of this approach is that the NCBI genome datasets and EMP 16s rRNA gene amplicon sequencing datasets are not geographically linked, and these genomes may not be representative of the 16s rRNA gene data. The abundance of each type was calculated by summing the abundance of all overlapping genera belonging to the corresponding type in different samples. The statistical analyses were performed using R v4.1.1.

### Isolation of thiamine-auxotrophic strains

The strains were isolated from soils by dilution separation methods on Luria–Bertani (LB) agar, and the thiamine auxotrophy of the obtained isolates were assayed using mineral salt medium (MSM) supplemented with additional carbon and nitrogen sources (thiamine-free medium, Supplementary methods). The purpose of adding the additional carbon and nitrogen sources was to provide the necessary nutrients for potential polyauxotrophs. Thiamine-auxotrophic isolates were identified if they were unable to grow after depleting all stored thiamine, but resumed growth after the addition of exogenous thiamine. *Escherichia coli* and its thiamine-auxotrophic mutant Δ*thiE* were used as controls. The detailed methods are proved in Supplementary methods.

### DNA stable isotope probing experiment

We investigated the degradation efficiency of bromoxynil by microbiota from two different pretreated soils. The detailed methods for soil pretreatment are proved in Supplementary methods. Normal (^12^C) and ^13^C-labeled bromoxynil were used in the experiment. A 50 ml sterilized conical flask was filled with 20 ml of MSM and 1 g of pretreated soils, and the initial concentration of bromoxynil was set to 0.07 mM. All cultures were incubated at 30°C and 180 rpm. Total DNA was extracted using the FastDNA Spin Kit (Solon, USA) according to the manufacturer’s instructions. The ultrahigh density centrifugation layering was performed as previously described [28]. A total of 14 fractions were collected by a fraction recovery system (Beckman Coulter), and the fractionated DNA was purified according to the method as previously described [51]. The purified DNA derived from fractions 5–12 in the DNA-SIP assay was further used to analyze the bacterial composition to identify bromoxynyl-degrading strains. Amplicon sequencing was performed on a MiSeq System (Illumina) according to the standard protocols at Biozeron Biotechnology Co. (Shanghai, China). The details of SIP experiment and data analysis are described in Supplementary methods.

### Microbial community model construction and optimal community combination analysis

The microbial community models were constructed based on the single-species model of each strain. For microbial community simulation, different combinations of strains were constructed using COBRAToolbox-3.0 [26]. The parsimonious FBA (pFBA) was used to calculate the fluxes, optimize the biomass function, and minimize the flux of each nutrient exchange reaction [52]. The detailed methods for construction of the single-species and community models are proved in Supplementary methods.

### Testing the computational predictions

To test the computational predictions of bromoxynil-degrading efficiencies by different consortia experimentally, bromoxynil-degrading rates by strain 7D-2 and/or different strain combinations in both MSM and *in situ* soils were measured. The setting of strain combinations and proportions were consistent with those in community model construction. The predicted metabolic interactions among strains were experimentally tested by detecting secreted metabolites in cocultures (no secreted metabolite was in the initial medium of cocultures) and supporting the growth of strains by the secreted metabolites [19]. The consortium (7D-2 + N1 + A1) was used to detect secreted metabolites by different strains. To verify the predictions that strain 7D-2 could use thiamine provided by other strains, we tested whether extra thiamine could improve the growth of strain 7D-2. Similarly, the supporting growth of strains by the secreted metabolites provided by other strains was tested by comparing the growth of strains cultured in medium supplemented with corresponding secreted metabolites vs. those without supplementary secreted metabolites. The detailed methods are proved in Supplementary methods.

### Quantitative PCR analysis

To assess the expression of thiamine-related genes, strains 7D-2 and N1 were individually cultured or cocultured in MSM supplemented with broximial (0.07 mM). After 6 and 11 h of cultivation, the bacterial cells were harvested for RNA extraction. Total RNA was extracted with a Bacteria RNA Extraction Kit (R403-01; Vazyme, China) and reverse-transcribed into cDNA using HiScript III-RT SuperMix for quantitative PCR (qPCR) (R323–01; Vazyme, China) following the manufacturer’s instructions. qPCR was performed with ChamQ SYBR qPCR Master Mix (Q311-02; Vazyme, China) and a Mastercycler ep realplex system (Eppendorf, Germany). *gyrA* was used as the internal control. The relative gene expression levels were calculated using the 2^–∆∆Ct^ method [53]. The primers used are listed in Table S1.

## Results

### Detecting key functional strains involved in bromoxynil degradation in soil microbiota by stable isotope probing

We used a bioaugmentation experiment to assess its impact on shaping the *in situ* microbiota toward developing a functional microbiota capable of degrading pollutants. The soils were amended with (i) microbial consortium (strains X-1 and 7D-2, X-1 + 7D-2) inoculation and (ii) the combination of herbicide (bromoxynil) application and microbial consortium (X-1 + 7D-2) inoculation. The details of the treatments are described in Supplementary methods. Although the initial microbiota in soils was incapable of degrading bromoxynil, the microbiota in amended soils could degrade bromoxynil efficiently (Fig. S2). In addition, different degrading efficiencies of bromoxyni by the two treatments implied variations in microbiota and/or metabolic interactions among strains in amended soils (Fig. S2), as the function of microbial communities relies on their composition and interactions among strains. To clarify the effects of bioaugmentation on the microbiota in soils, 16S rRNA genes and internal transcribed spacer (ITS) genes of the microbiota were sequenced by high-throughput sequencing (Fig. S2). Significant shifts were detected in bacterial rather than fungal communities based on the alpha and beta diversity analysis (Fig. S2). Thus, we focused on the bacterial community by SIP.

**Figure 1:**
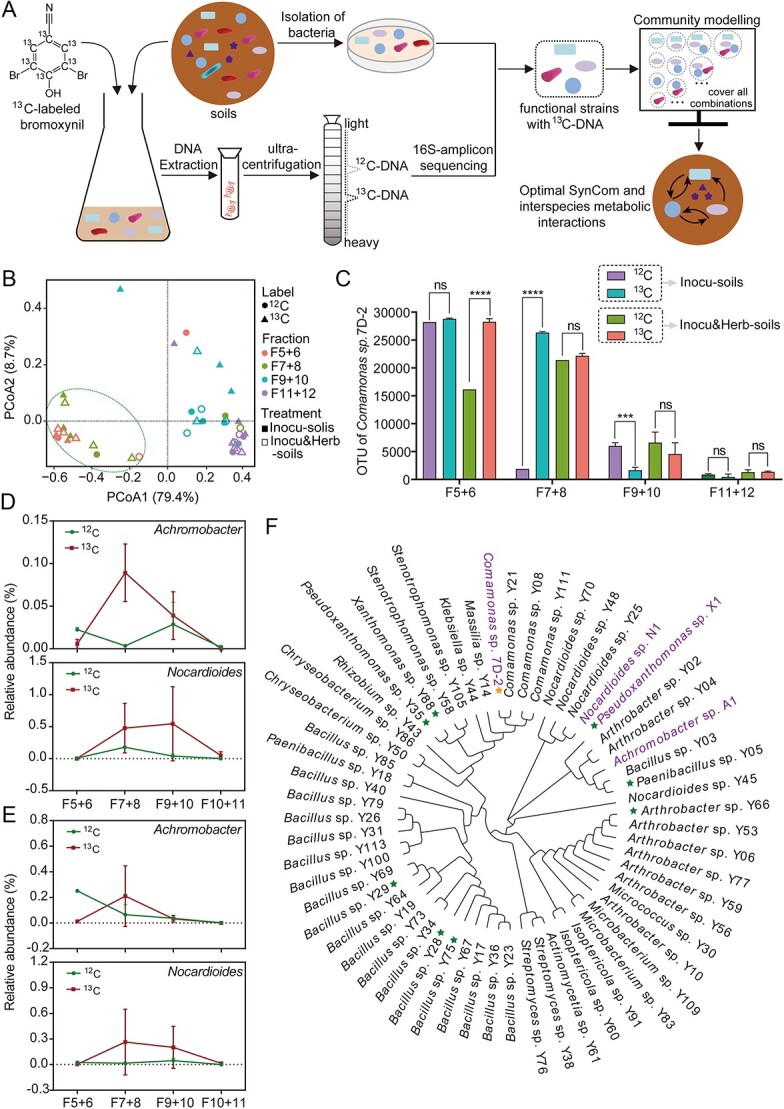
Detecting functional keystones by SIP; (A) workflow of the SIP-metabolic modeling method; briefly, the functional keystones were identified by SIP, which were further obtained by strain isolation from soils; the metabolic models of bacterial communities consisting of different functional keystones were constructed to explore the metabolic interactions; (B) PCoA with Bray–Curtis distances showing differences in bacterial communities between fractions 5 and 8 from ^13^C samples and others; (C) the number of operational taxonomic units (OTUs) corresponding to strain 7D-2, which is the only strain in soils capable of degrading and assimilating bromoxynil, showing that DNA in fractions 5–8 was labeled with ^13^C; (D, E) relative abundances of *Achromobacter* and *Nocardioides* in inoculation (inocu-soils, D) and inoculation-herbicide treatments (incobu-herb-soils, E), showing that the two genera were significantly enriched in ^13^C samples; (F) phylogenetic distribution of the 61 isolated strains from soils; the strains capable of degrading BO to bromoxynil or the strain 7D-2 with bromoxynil-degrading ability are labeled with stars; the strains 7D-2, X-1, N1, and A1 are used for metabolic modeling.

To identify bacteria involved in bromoxynil degradation in bacterial communities, the amended soils were cultured in medium with normal (^12^C) or ^13^C-labeled bromoxynil, and genomic DNA was extracted and fractionated by isopycnic gradient centrifugation (Fig. 1A). The buoyant density gradually decreased with the increase in the number of fractions (Fig. S3). The 16S rRNA genes of different fractions (fractions 5–12 with adjacent pairwise merging) from ^12^C and ^13^C samples were analyzed by high-throughput sequencing. In principal coordinates analysis (PCoA) analysis, the communities of fractions 5–8 in the ^13^C samples were clustered together in both the inoculation and inoculation–herbicide treatments (Fig. 1B). The abundance of 7D-2 (the degrader of bromoxynil) in the ^13^C samples was significantly higher than that in ^12^C samples in fractions 5–6 (inoculation–herbicide treatment) and 7–8 (inoculation treatment, Fig. 1C). In addition, for both soils with inoculation and inoculation–herbicide treatments, the 7D-2 abundances in fractions 5–8 were significantly higher than others in ^13^C samples (Fig. 1C). Therefore, the DNA in fractions 5–8, labeled with ^13^C, was referred to as “heavy DNA” and used for further analysis.

Differences in the community composition of fractions 5–8 between the ^12^C and ^13^C samples were analyzed by the Kruskal–Wallis H test (Fig. 1D and E). For both inoculation and inoculation–herbicide treatments, the relative abundances of *Achromobacter* and *Nocardioides* were higher in fractions 7–8 of ^13^C samples than in ^12^C samples (Fig. 1D and E). Specifically, the relative abundances of *Nocardioides* in fractions 7–8 of ^13^C samples were 0.48% and 0.26% for inoculation and inoculation–herbicide treatments, respectively, which were 0.18% and 0.02% in ^12^C samples, respectively (Fig. 1D and E). The results indicated that strains of *Achromobacter* and *Nocardioides* were involved in the assimilation of ^13^C-labeled bromoxynil, which were defined as keystone genera related to bromoxynil degradation.

In parallel to community analysis, we isolated strains related to bromoxynil degradation in amended soils, and 61 typical strains were isolated for bromoxynil-degrading ability detection (Fig. 1F). Among them, eight strains showed the ability to degrade BO into bromoxynil (Fig. 1F). However, only the exogenously inoculated strain 7D-2 could further degrade bromoxynil. In addition, two strains, N1 and A1, were classified as *Nocardioides* sp. and *Achromobacter* sp., respectively (Fig. 1F). According to the SIP results, the strains N1 and A1, combined with the exogenous inoculated strains 7D-2 and X1, were designed as key functional strains that were used for further modeling analysis.

### Simulation-based SynCom design for enhanced bromoxynil degradation

We constructed GSMMs for strains N1, A1, 7D-2, and X1, which were manually curated based on experimental data (Table S2, Supplementary Data). According to the experimental results, all four strains could grow separately in MSM with glucose and NH_4_^+^ (MSM-glucose-NH_4_^+^) as carbon and nitrogen sources (Fig. S4, Table S3). We used MSM-glucose-NH_4_^+^ medium to simulate the growth of the four strains. If a draft model failed to produce biomass under conditions where strain growth was experimentally observed, it indicated missing metabolic reactions in the model. These missing reactions could be due to incomplete genome annotation, requiring manual addition to the model. For model curation, the missing metabolic reaction was identified and added to the initial model manually until the biomass was reproduced. In addition, as 7D-2 can grow with bromoxynil as the only carbon and nitrogen source, the reactions involved in the bromoxynil-degrading pathway were added to the 7D-2 model. We also fine-tuned the growth simulation based on the literature and additional annotation of their phylogenetic relatives to correctly represent their biological characteristics. The curated GSMMs for strains 7D-2, X-1, A1, and N1 had 1605, 1481, 1650, and 1641 reactions and 1609, 1506, 1709, and 1638 metabolites, respectively (Table S2).

**Figure 2:**
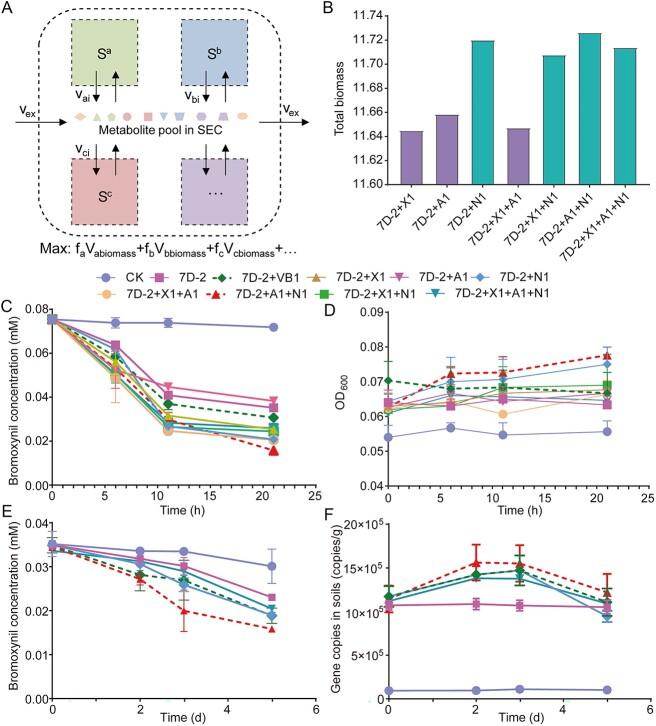
Simulations and experimental validations of bromoxynil degradation and bacterial growth performances; (A) schematic representation of compartmented community models; briefly, each single model is treated as a separate compartment to simulate an independent cell where reactions occur, and a “shared extracellular compartment” (SEC) is set to simulate the extracellular environment, allowing exchange of metabolites among species within the metabolite pool in the SEC; Sa, Sb, and Sc represent the reaction stoichiometry (S matrix) of cells a, b, and c, respectively; *v*_ai_ is the flux of reaction i in cell a; *v*_ex_ is the flux of exchange reactions; and *f*_a_ is the fraction of cell a in the community’s biomass; the objective function is maximizing the total biomass of the community; (B) predicted total biomass of different combinations of functional strains; (C–F) experimental validation of simulations by experiments; the bromoxynil-degrading efficiency of different combinations was tested in medium (C) and soils (E) supplemented with bromoxynil; (D) the growth of different communities in medium with bromoxynil; (F) the abundance of strain 7D-2 in soils revealed by gene copy numbers obtained by qPCR; VB1, vitamin B1 (i.e. thiamine).

**Figure 3:**
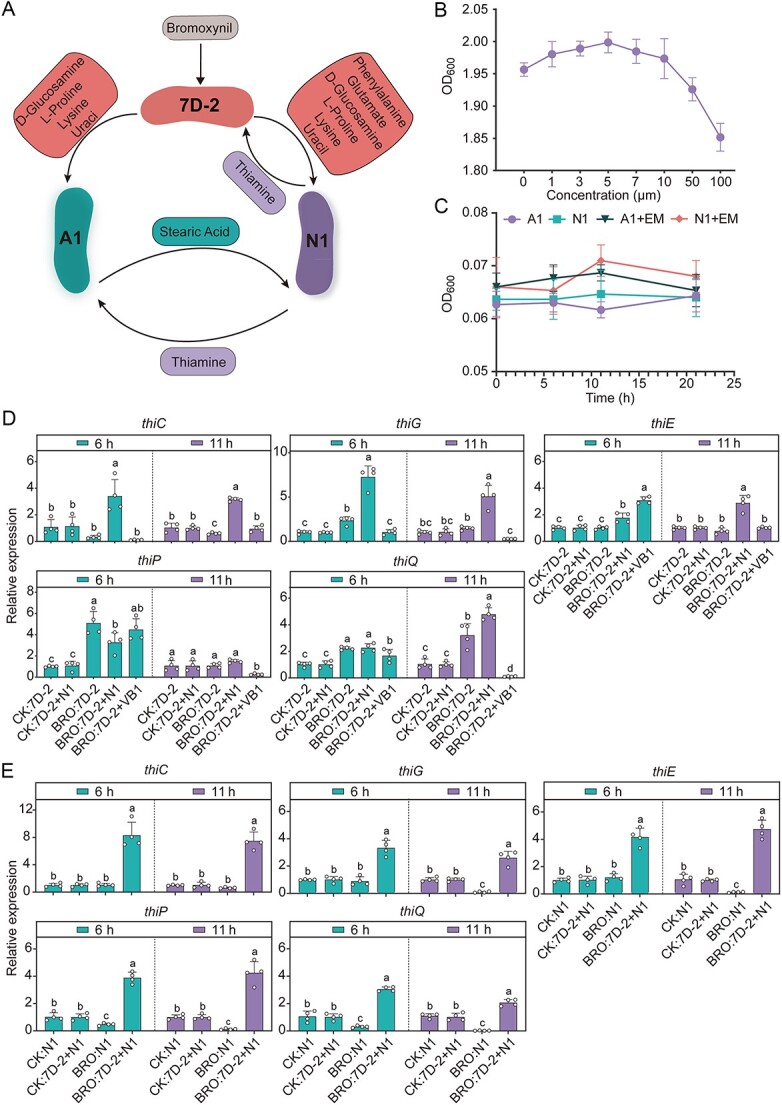
Prediction and validation of metabolic interactions in the optimal SynCom; (A) predicted exchange fluxes in medium with bromoxynil as the only carbon and nitrogen source; (B) the growth of 7D-2 cells cultured in medium with bromoxynil and different concentrations of thiamine; (C) the growth of strains N1 and A1 in medium containing bromoxynil as the sole nitrogen and carbon source and the same medium supplemented by EMs; (D, E) the relative expression of genes involved in thiamine biosynthesis and transportation in strains 7D-2 (D) and N1 (E); the measurements were performed for both single and cocultures of 7D-2 and N1 in medium with or without bromoxynil; the cultures in medium without bromoxynil were used as control (CK); VB1, vitamin B1 (i.e. thiamine); BRO, bromoxynil.

We further simulated the performances of different combinations of 7D-2 with others to obtain communities that were able to enhance bromoxynil degradation in nitrogen-free MSM with bromoxynil as the only carbon and nitrogen source (MSM-bromoxynil medium) (Fig. 2A and B). A total of seven combinations were set, and 7D-2 + N1 + A1 showed the maximal biomass (Fig. 2B) that was identified as the “bromoxynil-degrading SynCom.” To explore metabolic interactions in 7D-2 + N1 + A1, we predicted fluxes of all reactions during the bromoxynil-degrading process by this community in MSM-bromoxynil medium. The simulation predicted that 7D-2 provided lysine, uracil, proline, and D-glucosamine for A1 and glutamate, lysine, phenylalanine, uracil, proline, and D-glucosamine for the N1. In return, N1 provided thiamine for 7D-2 and A1, while strain A1 provided stearic acid for N1 (Fig. 3A).

### Experimental validation of simulation-based predictions

We measured the growth and bromoxynil-degrading efficiency of different communities used for modeling in both flask and *in situ* soils to test the performances of different combinations (Fig. 2C–F). The experimental results were consistent with simulations obtained by modeling. First, the combination of 7D-2 + N1 + A1 grew fastest in MSM-bromoxynil medium (Fig. 2D). In addition, the bromoxynil-degrading efficiency of 7D-2 + N1 + A1 was much higher than that of 7D-2 alone or other combinations in both MSM-bromoxynil medium and soils (Fig. 2C and E). Furthermore, the cell numbers of 7D-2 (revealed by copy numbers of *Bxn2*, which was measured by qPCR) were much higher in soils treated with 7D-2 + N1 + A1 than in other soils (Fig. 2F).

To experimentally verify predicted metabolic interactions in 7D-2 + N1 + A1, we first detected secreted metabolites in cocultures of 7D-2 + N1 + A1 in the MSM-bromoxynil medium to verify predicted secretions (Fig. S5, Table S4). We then tested the support of stain growth with the secreted metabolites to verify predicted assimilation (Fig. 3B and C). All exchanged metabolites (EMs) were successfully detected by liquid chromatography–mass spectrometry (LC-MS) in the coculture of 7D-2 + N1 + A1, except for D-glucosamine, which might be due to its rapid utilization by cells. Meanwhile, the EMs could improve the growth of strains 7D-2, A1, and N1 (Fig. 3B and C), showing the support of stain growth with the secreted metabolites. These results showed that the experimental results were consistent with the model predictions.

### Thiamine-mediated metabolic interactions in the bromoxynil-degrading SynCom

Both the strains 7D-2 and N1 could grow separately in MSM-glucose-NH_4_^+^ medium (Fig. S4), showing both strains were thiamine-prototrophic. However, in the simulation, 7D-2 obtained thiamine from the N1. Thus, we tested the performance of 7D-2 in the bromoxynil-degrading process supplemented with thiamine. In both MSM-bromoxynil medium (Fig. 3B) and *in situ* soils (Fig. 2F), the addition of thiamine promoted 7D-2 growth and improved the bromoxynil-degrading efficiency (Fig. 2C and E). Thiamine also increased the cell number of 7D-2 in soils (Fig. 2F).

The expression levels of thiamine-related genes in 7D-2 and N1 were assessed using qPCR to verify that thiamine biosynthesis and transport were enhanced during bromoxynil-degrading processes (Fig. 3D and E). The genes *thiC*, *thiG*, and *thiE* were used because they are key genes for thiamine biosynthesis, while *thiP* and *thiQ* were used because they are involved in thiamine transport. For both strains, the highest expression of *thiC*, *thiG*, and *thiE* was detected in cocultures of 7D-2 and N1 in MSM-bromoxynil medium (Fig. 3D and E). Compared to the cocultures in medium with bromoxynil, significantly lower expression levels of the three genes were detected in single cultures (no matter in medium with or without bromoxynil) and cocultures in medium without bromoxynil (Fig. 3D and E). Consistently, the addition of exogenous thiamine decreased the expression of *thiC*, *thiG*, and *thiE* in 7D-2 cells (Fig. 3D).

For thiamine-transport genes, significantly increased expression of *thiQ* at 11 h was detected in 7D-2 cocultured with N1 in medium with bromoxynil compared to those in single cultures or cocultures without bromoxynil (Fig. 3D). Similarly, significantly higher expression of *thiP* and *thiQ* was detected in N1 in cocultures with 7D-2 in medium with bromoxynil compared to others (Fig. 3E). Together, these results showed that thiamine biosynthesis and transportation in both 7D-2 and N1 were specifically induced by cocultures and bromoxynil in environments, indicating different strategies of thiamine-related metabolism were used by single strains and the cocultured community. These results provided evidence of the involvement of thiamine-mediated metabolic interactions between strains 7D-2 and N1 in the collective response to bromoxynil application.

### Exploring thiamine-mediated metabolic interactions in soil bacteria

**Figure 4:**
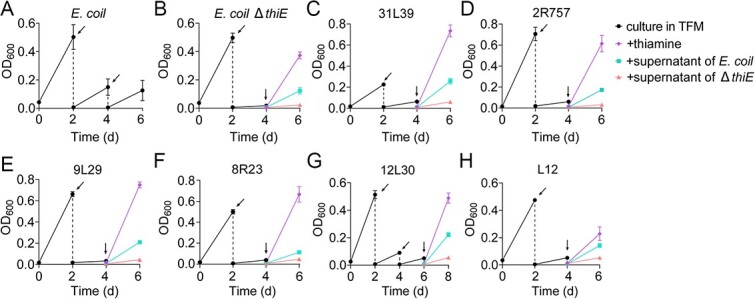
Thiamine dependence of auxotrophs on prototrophs; (A) the thiamine-prototrophic strain *E. coli*; (B) the thiamine-auxotrophic strain *E. coli* K-12 Δ*thiE*; (C–H) the thiamine-prototrophic strains isolated from soils, including *Microbacterium* sp. 31 L39 (C), *Massilia* sp. 2R757 (D), *Microbacterium* sp. 9 L29 (E), *Massilia* sp. 8R23 (F), *Microbacterium* sp. 12 L30 (G), and *Terrabacter* sp. L12 (H); all the isolates were firstly cultured in a thiamine-free medium (TFM), and dilution transfers (oblique arrows) were performed to consume the stored thiamine in the cells of the isolates; to restore growth of the isolates, during the last dilution transfer (vertical arrows), the cultures of thiamine-auxotrophic isolates were inoculated into the culture supernatant of *E. coli* or *E. coli* K-12 Δ*thiE*, or new TFM supplemented with exogenous thiamine.

The SIP-metabolic modeling results pointed out thiamine-mediated metabolic interactions between strains 7D-2 and N1. We further investigated whether thiamine-mediated metabolic interactions occur among other bacterial strains from soils, focusing on the thiamine auxotrophs’ reliance on prototrophs. To isolate thiamine-auxotrophic strains from agricultural soils, we utilized *E. coli* K-12 Δ*thiE*, a thiamine-auxotrophic strain that can only grow in the presence of exogenous thiamine, as the indicator strain (Fig. 4, Fig. S6). Six thiamine-auxotrophic strains were isolated from the soil, exhibiting a similar growth pattern to the strain K-12 Δ*thiE*: no growth in a defined medium lacking thiamine and restored growth by addition of exogenous thiamine (Fig. 4B, Fig. S6). In contrast, the wild-type strain *E. coli*, a thiamine-prototrophic strain, grew well in the same defined medium without thiamine (Fig. 4A). Additionally, four thiamine-prototrophic strains were isolated from soils (Fig. S7), and we tested the thiamine-mediated metabolic interactions between the thiamine-auxotrophic and -prototrophic strains (Fig. 4C–H). As expected, the culture supernatant of *E. coli* (grown in thiamine-free medium) restored the growth of the strain K-12 Δ*thiE* and the other six thiamine-auxotrophic strains in the defined medium lacking thiamine (Fig. 4C–H). Similar growth patterns were observed between the thiamine-auxotrophic and -prototrophic strains from soils (Fig. S7), suggesting that thiamine-prototrophic bacteria in soils could supply thiamine for the growth of thiamine-auxotrophic bacteria. To further explore the prevalence of thiamine-mediated metabolic interactions in bacterial communities, and to understand how these interactions are involved in bacterial evolution and community assembly, we investigated the phylogenetic and global distribution of thiamine-auxotrophic and -prototrophic bacteria based on bacterial genomes from NCBI and soil samples from EMP.

### Phylogenetic distribution of bacteria harboring the thiamine biosynthetic pathway

**Figure 5:**
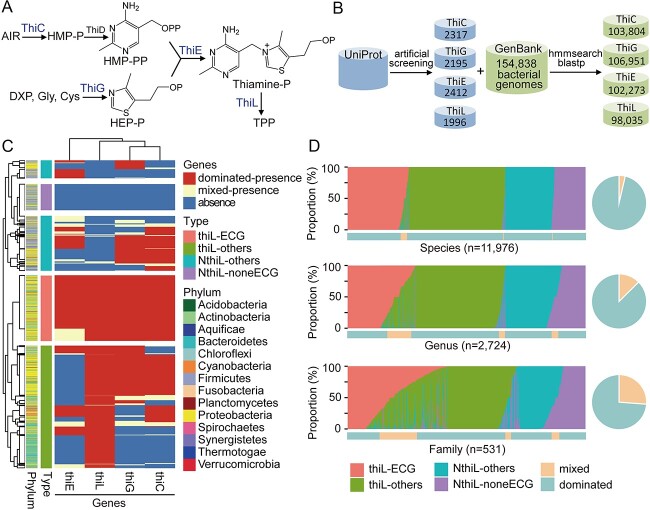
Phylogenetic distribution of the thiamine biosynthetic pathway; (A) overview of the thiamine biosynthesis pathway in bacteria; Abbreviations: AIR, 5-aminoimidazole ribonucleotide; HMP, 4-amino-2-methyl-5-hydroxymethylpyrimidine; HEP, hydroxyethylthiazole; DXP, 1-deoxy-D-xylulose 5-phosphate; TPP, thiamine pyrophosphate; (B) workflow used to identify thiamine biosynthetic genes; (C) the presence/absence pattern of the four key genes divided bacteria into four thiamine biosynthetic types at the genus level; the four types are as follows: thiL-ECG, taxa with all four genes; thiL-others, taxa with *thiL* but lacking any of the genes *thiC*, *thiE*, or *thiG*; NthiL-noneECG, taxa without any of the four genes; and NthiL-others, taxa without *thiL* but having at least one of *thiC*, *thiE*, and *thiG*; the genus containing genomes with specific genes (i.e. presence) and the percentage of presence ≥75% were considered dominant, and those <75% and >25% were treated as mixed, while those ≤25% were absent; (D) relative abundance of four types of thiamine biosynthetic patterns at different taxonomic levels.

**Figure 6:**
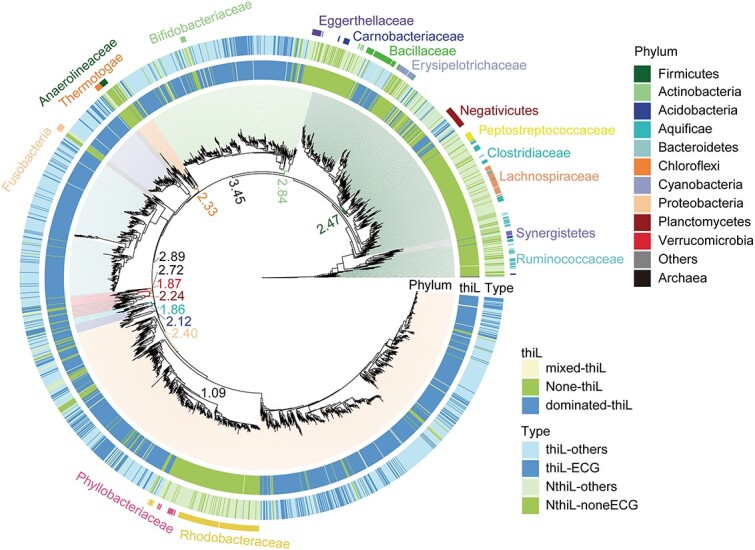
The reference phylogenetic tree of major lineages of bacteria at the genus level; the phylogenetic tree was reconstructed from the concatenate alignment of 20 universally conserved orthologous proteins; the colored numbers refer to the divergence times (billions of years) of the corresponding lineages, which were derived from Figure S9; the definitions of the four types and three thiL patterns are the same as in Figure 5.

To explore potential phylogenetic distributions of bacteria harboring thiamine-biosynthetic pathways and thiamine-mediated metabolic interactions between community members, the key genes involved in thiamine biosynthesis were analyzed, and then the thiamine-auxotrophic and thiamine-prototrophic bacteria were identified according to the completeness of the thiamine biosynthetic pathway (Fig. 5). In total, 154 838 bacterial genomes from NCBI, covering 11 976 species, 2724 genera, and 531 families, were analyzed to collect homologs of the key genes, i.e. *thiL*, *thiE*, *thiC*, and *thiG*, in the thiamine biosynthetic pathway (Fig. 5A and B). A total of 103 804, 106 951, 102 273, and 98 035 genomes with *thiC*, *thiG*, *thiE*, or *thiL* were identified, respectively (Fig. 5B). Considering the taxonomic redundancy of these genomes, we further analyzed the phylogenetic distributions at the genus level (Fig. 5C). The presence/absence pattern of the four genes divided bacteria into two main groups: strains with and without *thiL* (Fig. 5C). TPP is the biologically active form of thiamine, and ThiL catalyzes the transformation of thiamine monophosphate to TPP, so this conversion is essential in cells for thiamine to be used in metabolic processes. Therefore, the two groups could be recognized as thiamine-needed (the group with *thiL*) and thiamine-unrequired (the group without *thiL*) bacteria, respectively. The thiamine-needed bacteria were further divided into two main types (Fig. 5C): strains harboring all four essential genes (thiL-ECG) were classified as thiamine prototrophs, while strains possessing *thiL* but lacking any of the genes *thiC*, *thiG*, or *thiE* (thiL-others) were classified as thiamine auxotrophs. Similarly, the thiamine-unrequired bacteria were divided into two main types (Fig. 5C): NthiL-noneECG, strains lacking all four genes; and NthiL-others, strains lacking *thiL* but possessing at least one of *thiC*, *thiG*, and *thiE*. Most taxa were dominated by only one type, especially at the species (96%) and genus (87%) levels (Fig. 5D, Fig. S8). The mixed types were mostly mixed between thiL-ECG and thiL-others (Fig. 5D), showing the possibility of transformations between thiamine prototrophs and -auxotrophs and rare transformations between thiamine-needed and -unrequired bacteria. This conclusion was also supported by the phylogenetic distribution of *thiL* and the four types (Fig. 6). Generally, the thiamine-needed and -unrequired bacteria were clustered into different clades, while the thiamine prototrophs and -auxotrophs were distributed in the same clusters (Fig. 6).

Most of the thiamine-unrequired bacteria were anaerobic, such as *Ruminococcaceae*, *Synergistetes*, *Lachnospiraceae*, *Clostridiaceae*, *Peptostreptococcaceae*, *Erysipelotrichaceae*, *Carnobacteriaceae*, *Eggerthellaceae*, *Bifidobacteriaceae*, *Anaerolineaceae*, *Thermotogae*, *Fusobacteria*, *Phyllobacteriaceae*, and *Rhodobacteraceae* (Fig. 6), consistent with the essential function of thiamine in aerobic metabolism. The basal taxa in the phylogenetic tree, including *Ruminococcaceae*, *Synergistetes*, *Lachnospiraceae*, *Clostridiaceae*, and *Peptostreptococcaceae*, were thiamine-unrequired and anaerobic, while most of the derived clades were aerobic and thiamine-needed (Fig. 6), implying the evolution of aerobic thiamine-needed bacteria from anaerobic thiamine-unrequired ancestors. To verify this hypothesis, a time-calibrated phylogenetic tree was constructed to estimate the divergence time of the main clades of bacteria (Fig. S9). A thiamine-needed lineage (including *Bacillaceae* and *Negativicutes*) was detected in *Firmicutes*, originating from 2.47 Bya (Fig. 6, Fig. S9). In addition, the origin time of the main thiamine-needed bacterial clades ranged from 1.87 to 2.84 Bya, which were before or during the Paleoproterozoic period, with composite 95% confidence intervals overlapping with the GOE (Fig. 6, Fig. S9). These findings implied that thiamine-needed bacteria evolved in response to the rise in oxygen concentration due to GOE. In contrast, the origin time of the thiamine-unrequired lineage in *Proteobacteria* (including *Phyllobacteriaceae* and *Rhodobacteraceae*) was 1.09 Bya (Fig. 6, Fig. S9), indicating their independent evolution from the thiamine-needed ancestors, which might arise from gene loss of *thiL*.

### Global geographic distribution and diversity of thiamine-auxotrophic and thiamine-prototrophic bacteria

**Figure 7:**
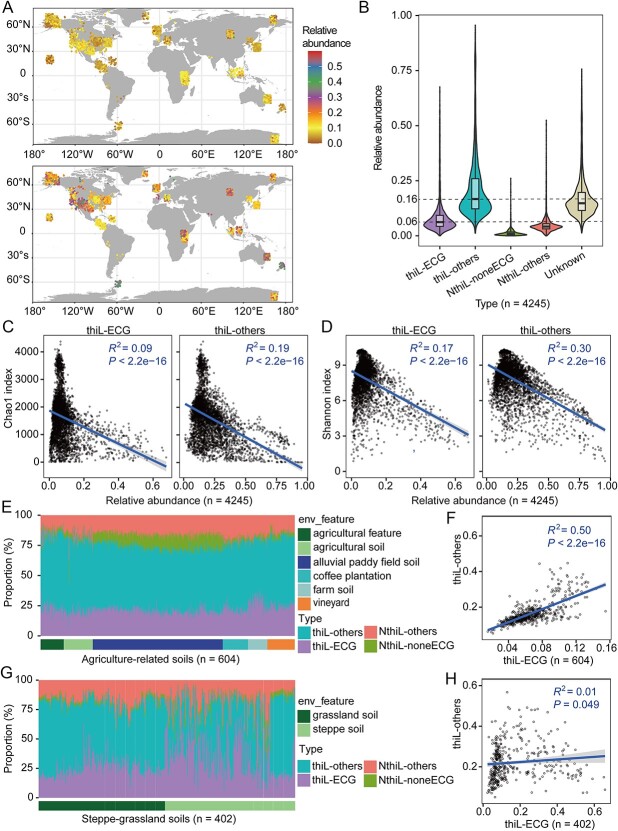
Distribution pattern of thiamine-auxotrophic and thiamine-prototrophic bacteria in global environments; (A) geographic and abundance distribution of thiamine-prototrophic (above, type: thiL-ECG) and thiamine-auxotrophic (below, type: thiL-others) bacteria in each sample; (B) average relative abundance of the four thiamine biosynthetic types based on the 4245 soil samples shown in Figure 7A; genera detected by 16S rRNA gene data but lacking genomes in NCBI were categorized as “unknown” type; (C, D) the relationship between the Chao1 (C) or Shannon (D) index of bacterial communities and relative abundances of thiamine prototrophs (thiL-ECG, left) or thiamine auxotrophs (thiL-others, right); (E, G) relative abundances of the four types within the agriculture-related (E) and steppe-grassland (G) soils; (F, H) the relationship between the relative abundances of thiamine auxotrophs and -prototrophs within agriculture-related (F) and steppe-grassland (H) soils; the definitions of the four types are the same as in Figure 5.

Thiamine-auxotrophic and -prototrophic bacteria were widely distributed on a global scale (Fig. 7A, Fig. S10). The relative abundances of thiamine prototrophs were between 0.2% and 67.8% (median = 6.0%), while those of thiamine auxotrophs were even higher, between 1.2% and 95.6% (median = 16.0%, Fig. 7A and B). Globally, there is a weak—but significant—relationship between latitude and the relative abundance of both thiamine-prototrophic and -auxotrophic bacteria (*P* < .001, Fig. S11). In addition, a significant correlation between the alpha diversity of the bacterial communities and the relative abundances of thiamine prototrophs or -auxotrophs was detected (*P* < 2.2e−16, Fig. 7C and D). These results indicated that thiamine-mediated metabolic interactions between thiamine prototrophs and -auxotrophs were globally distributed, affecting bacterial community diversity. We then focused on the habit of agriculture-related and steppe-grassland soils (Fig. 7E–H). The ratio of thiamine prototrophs and -auxotrophs was relatively stable in agriculture-related soils (Fig. 7E). Correspondingly, the relative abundance of thiamine prototrophs and -auxotrophs showed a significantly positive correlation (*R*^2^ = 0.50, *P* < 2.2e−16, Fig. 7F), showing the thiamine dependence of auxotrophs on prototrophs. In contrast, more variations in the ratio of thiamine prototrophs and -auxotrophs were detected in steppe-grassland soils (Fig. 7G), and the correlation coefficient between their relative abundance was low (*R*^2^ = 0.01, *P* = .049, Fig. 7H). In addition, for steppe-grassland soils, the relative abundance of thiamine prototrophs was significantly correlated with alpha diversity, but such a correlation was not detected for thiamine auxotrophs (Fig. S12). These results showed a weak thiamine dependence of auxotrophs on prototrophs in steppe-grassland soils. One possible explanation was that thiamine auxotrophs might obtain extra thiamine produced by plants [54], implying that plants could affect the thiamine-mediated metabolic interactions among bacteria.

## Discussion

A fascinating question is how early life on Earth, which primarily relied on anaerobic lifestyles, managed to survive and thrive in an oxygenated world after GOE and successfully evolved into aerobic lifestyles. Numerous studies have focused on the origin and evolution of oxygenic photosynthesis [55, 56], antioxidant systems [57], carbon fixation pathways [10], and aerobic electron transport chains [9–11], showing that these biological reaction modules were important for early life evolution. Our research showed strong coincidences of the origin of thiamine synthetic bacteria with the rising oxygen concentration at the early Earth, indicating that the thiamine-synthetic pathway might also be involved in the evolutionary transition from anaerobic to aerobic lifestyles. These findings complement the evolutionary process by which organisms adapt to aerobic environments on early Earth, i.e. the origin of aerobic bacteria depends not only on the emergence of the aerobic respiratory chain and antioxidant systems but also on other supporting systems, such as thiamine-related reactions. The findings improved our understanding of the origins and evolutionary processes of aerobic life.

The crucial roles of thiamine in aerobic metabolism and the evolutionary process of thiamine synthetic bacteria imply that thiamine-mediated metabolic interactions might be one of the fundamental factors affecting bacterial community diversity. In this study, we integrated high-quality genomes from NCBI with 16S rRNA gene amplicon sequencing data from EMP to identify thiamine-prototrophic and -auxotrophic bacteria, and to explore the impact of thiamine-mediated interactions on bacterial communities. This combined approach has been utilized in multiple studies [48–50]. Metagenomic sequencing, which allows for the recovery of metagenome-assembled genomes (MAGs) [58], has also been employed to study distributions of functional strains. Although metagenomic sequencing excels in discovering new microbes and functions, its drawbacks include high costs and limited MAG recovery (usually fewer than 100–1000 MAGs per soil sample). In contrast, the NCBI repository contains over 150 000 high-quality bacterial genomes, representing more than 11 000 species across 2000 genera. Using the combined methods, we detected both thiamine-auxotrophic and -prototrophic bacteria across all tested soil environments, although their abundances varied. The abundances of thiamine auxotrophs and -prototrophs were significantly correlated with diversity indices of the bacterial community. For most niches, the ratio of abundances of thiamine auxotrophs to -prototrophs remained stable, implying a potential “consumer–producer” relationship for thiamine between them in soils. Additionally, we showed that thiamine prototrophs supported the growth of thiamine auxotrophs from soils, consistent with previous findings that auxotrophs maintain their population well when cultivated together with prototrophic strains that provide them vitamins [59]. These are also consistent with the theory that certain costly metabolites (such as thiamine) serve as “public goods” that are produced by specific members of a community and shared by the whole community [60–62]. Although the proportion of thiamine-prototrophic and -auxotrophic bacteria identified through 16S rRNA gene amplicon sequencing does not directly confirm thiamine-mediated interactions in soils, the stable ratio suggests that thiamine auxotrophs likely depend on thiamine produced by thiamine prototrophs. Together, these results showed that thiamine-prototroph abundance and thiamine-mediated metabolic interactions were important factors affecting bacterial community assembly and diversity.

To verify thiamine-mediated metabolic interactions in soils, we newly proposed an SIP-metabolic modeling method. The key functional strains were first obtained based on SIP, and metabolic interactions among them were subsequently documented by modeling, which was further tested experimentally. By this method, we successfully detected the complex metabolic interactions in soils and first uncovered that thiamine was also a key compound mediating metabolic interactions among thiamine prototrophs, which were largely ignored before. Furthermore, we showed the EMs enhanced the growth of strains 7D-2, A1, and N1. These findings demonstrated that metabolic interactions facilitated the utilization of environmental carbon and nitrogen sources, such as bromoxynil, by the SynCom of 7D-2 + N1 + A1, leading to improved growth, and bromoxynil degradation capability. The expression of thiamine-synthetic and transport genes was strongly induced by cocultures of 7D-2 and N1 in medium with bromoxynil, while no or weak induction was detected when cultured singly with bromoxynil or cocultured without bromoxynil. This suggests that thiamine-mediated metabolic interactions within the SynCom occurred exclusively during cocultures involving bromoxynil degradation. These results suggest that thiamine-mediated metabolic interactions may represent an adaptation strategy by bacterial communities to cope with changing environmental conditions, such as bromoxynil treatment, at the community level.

We demonstrated that SIP-metabolic modeling was an effective tool with application potential for exploring bacterial metabolic interactions, especially for discovering new metabolic interactions. This method can also be utilized to identify pollutant-degrading strains and potential “helper” strains—those without pollutant-degrading capabilities but which enhance degradation rates by supporting degraders—to construct SynComs for bioremediation. These SynComs offer promising solutions for remediation of POPs. However, limitations of the SIP-metabolic modeling method should be acknowledged, largely due to the current state of technology. First, there are chances of failure in the isolation of functional strains detected by SIP from soils. Second, due to the complexity of the model construction and analysis process, automated tools are needed for more complex microbiomes. In the near future, new technologies increasing the capability of strain isolation and facilitating model construction and analyses will increase the applied range of SIP-metabolic modeling and make it an integral part of microbial community study and application.

## Supplementary Material

SupplementaryFigures_wrae157

SupplementaryTables_wrae157

SupplementaryMethods_wrae157

## Data Availability

The amplicon sequencing data have been deposited in the NCBI SRA under the accession number PRJNA1049311.
