# Supplementary material for: Bacterial growth and environmental adaptation via thiamine biosynthesis and thiamine-mediated metabolic interactions
Source: ISME J. 2024 Aug 12;18(1):wrae157. doi: 10.1093/ismejo/wrae157 (PMC11346370; doi:10.1093/ismejo/wrae157)
Supplement: SupplementaryFigures_wrae157 [file supplementaryfigures_wrae157.pdf]

Supplementary Figures

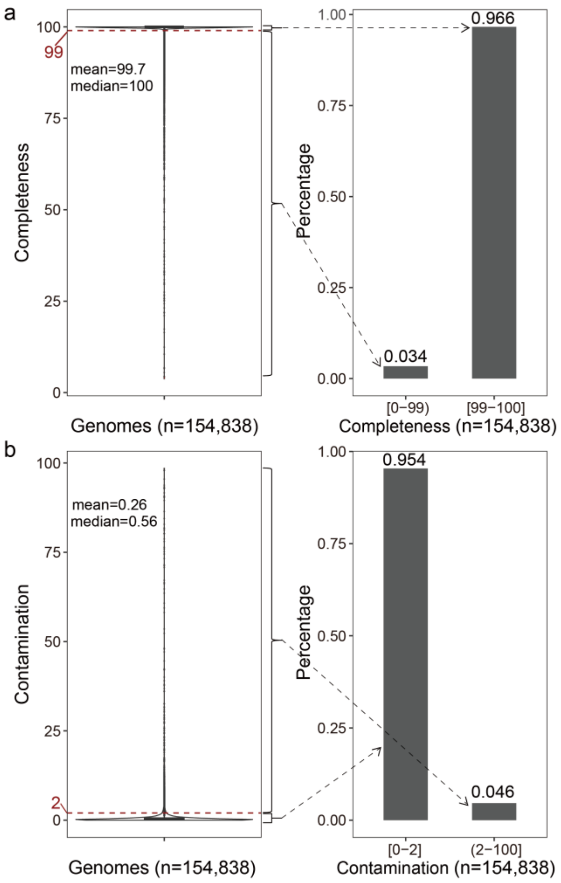

Fig. S1. The completeness and contamination of genomes. Violin plots on the left show the completeness (a) and contamination (b) of all genomes. Bar plots on the right show the percentage of genomes with completeness ≥99% and genomes with contamination ≤2%.

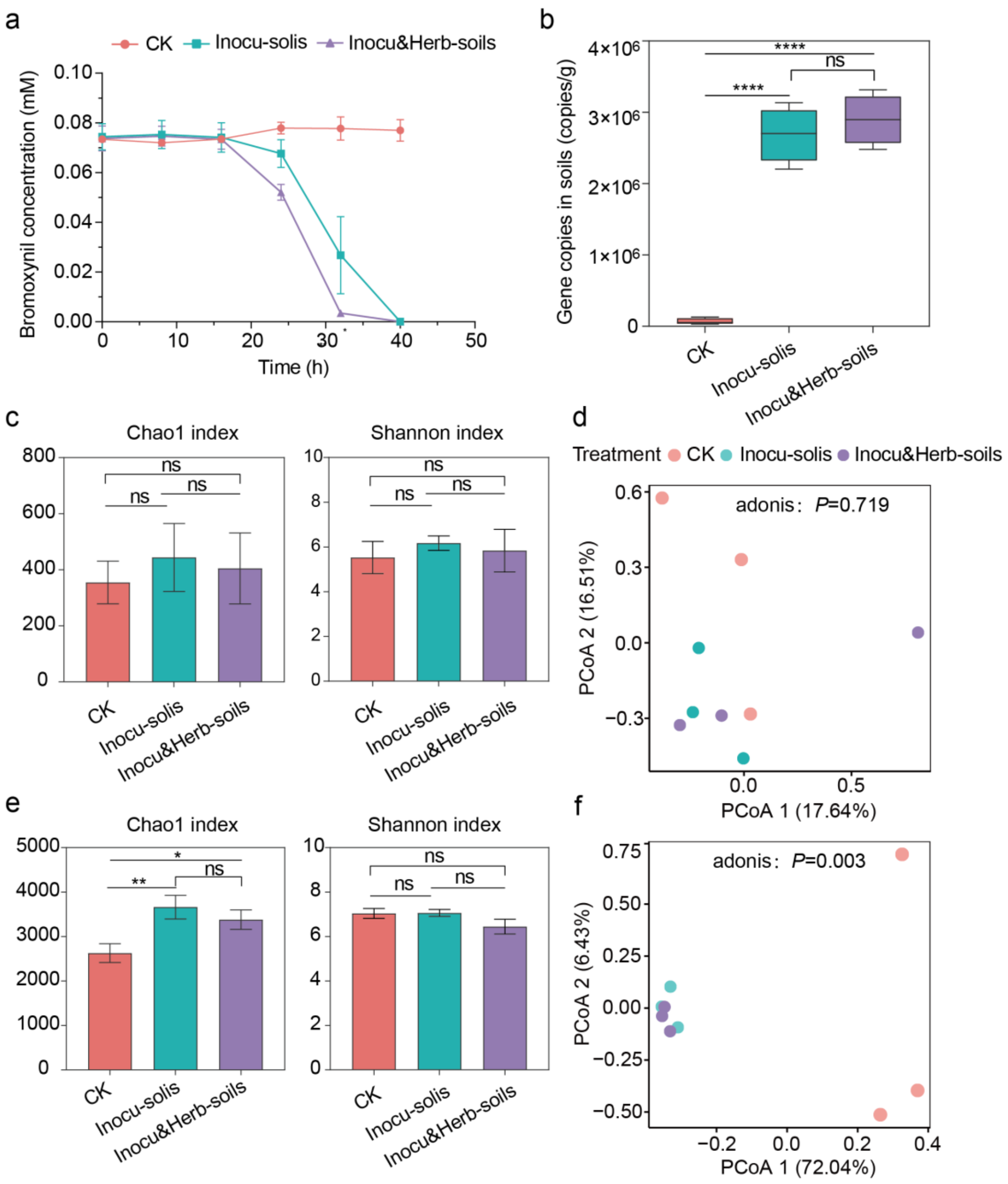

Fig. S2. Effects of bioaugmentation on BO degradation and microbial community shifting in BO-contaminated soils. a, The degradation ability of BO by microbiota from the inoculation (inocu-soils) and inoculation-herbicide (inocu&Herb-soils) treated soils. **b**, the abundance of strain 7D-2 in soils with different treatments revealed by gene copy numbers obtained by qPCR. **c-d**, No significant shifts of fungal community after treatments revealed by the alpha (c) and beta (d) diversity analysis. **e-f**, Significant shifts of bacterial community after treatments revealed by the alpha (e) and beta (f) diversity analysis.

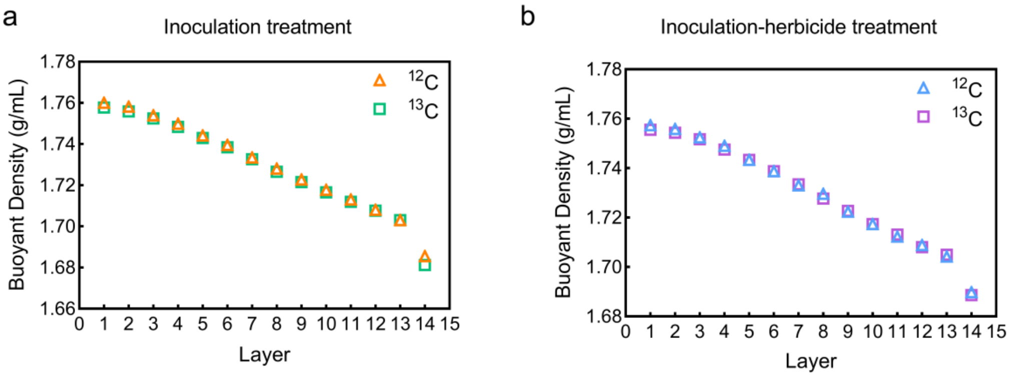

Fig. S3. The buoyancy density of ^{12}C and ^{13}C-labeled DNA in different layers. a, soils with inoculation treatment; **b**, soils with inoculation-herbicide treatment.

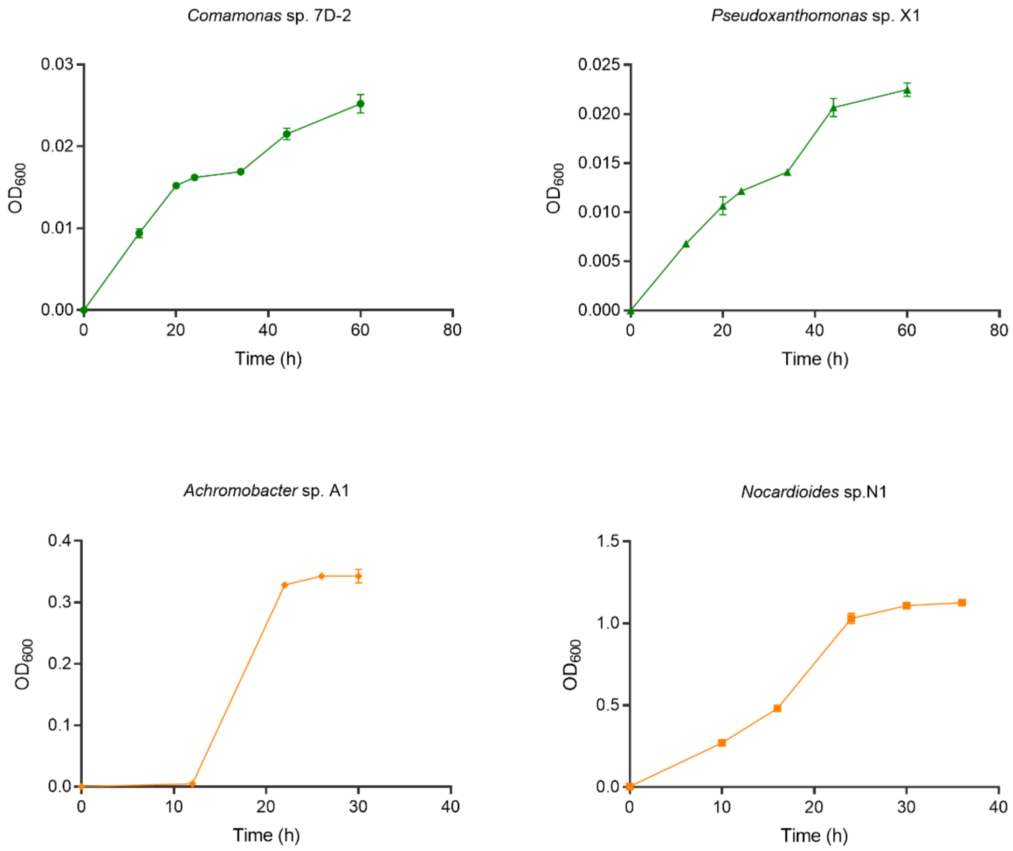

Fig. S4. The cell growth of keystones in minimal mineral medium with glucose and NH_4^+ as the carbon and nitrogen sources.

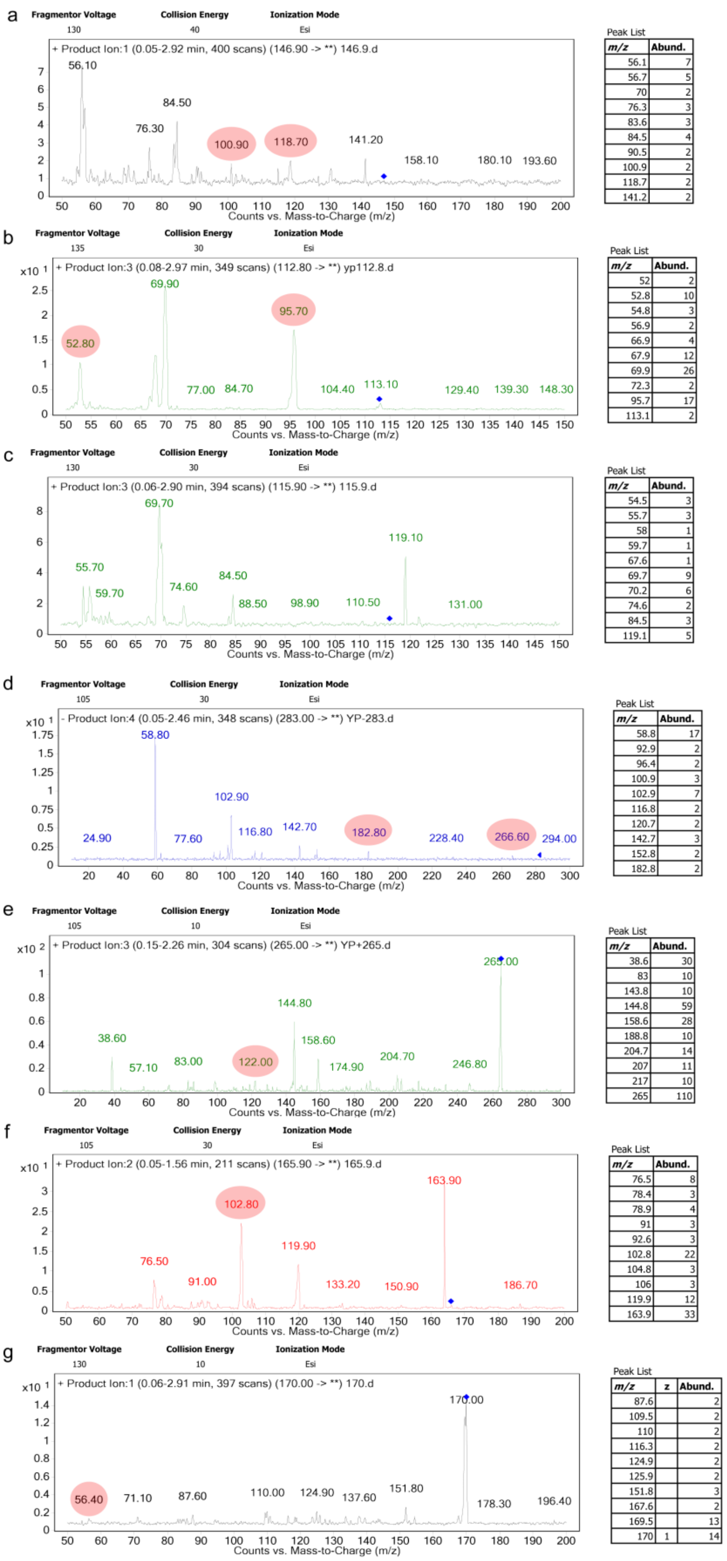

Fig. S5. Identification of predicted exchange metabolites (Fig. 3a) via LC-MS in a co-culture of strains 7D-2, A1, and N1 in a medium containing bromoxynil as a sole carbon and nitrogen source. a, lysine; **b**, uracil; **c**, proline; **d**, stearic acid; **e**, thiamine; **f**, phenylalanine; **g**, sodium glutamate. The fragment peaks of compounds in the second-order mass spectrum are shown. The quality spectrum of standard compound is shown in Table S4.

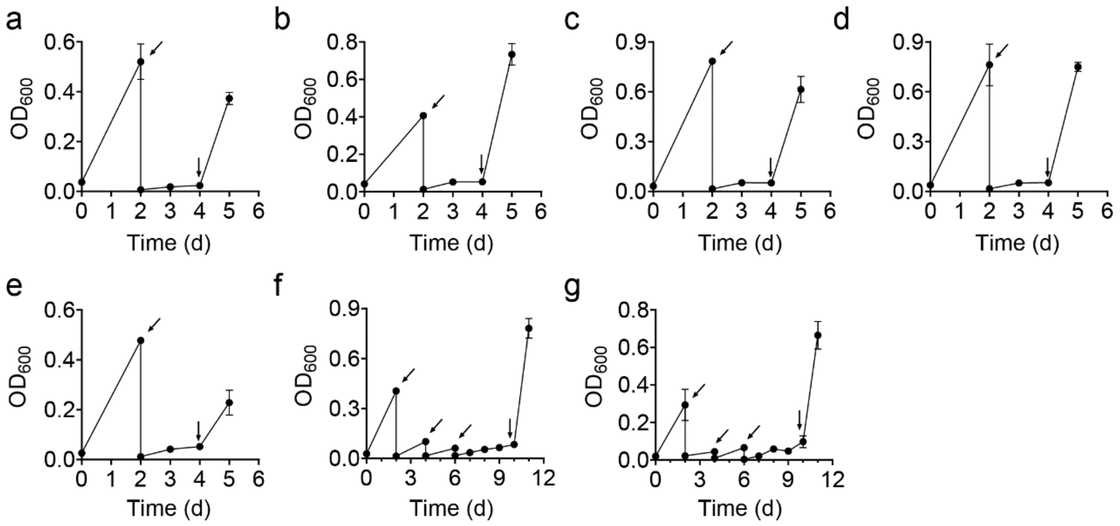

Fig. S6. Isolation of thiamine-auxotrophic strains. a, the thiamine-auxotrophic strain *E. coli* K-12 *ΔthiE*. **b-g**, The thiamine-prototrophic strains isolated from soils, including *Microbacterium* sp. 31L39 (**b**), *Massilia* sp. 2R757 (**c**), *Microbacterium* sp. 9L29 (**d**), *Terrabacter* sp. L12 (**e**), *Microbacterium* sp. 12L30 (**f**), and *Massilia* sp. 8R23 (**g**). The black arrows indicate the dilution transfers. Dilution transfers were performed to consume the stored thiamine in the cells of the isolates. To restore growth of the isolates, during the last dilution transfer (vertical arrows), the cultures of thiamine-auxotrophic isolates were inoculated into new thiamine-free medium supplemented with exogenous thiamine. All the isolates were cultured in a thiamine-free medium.

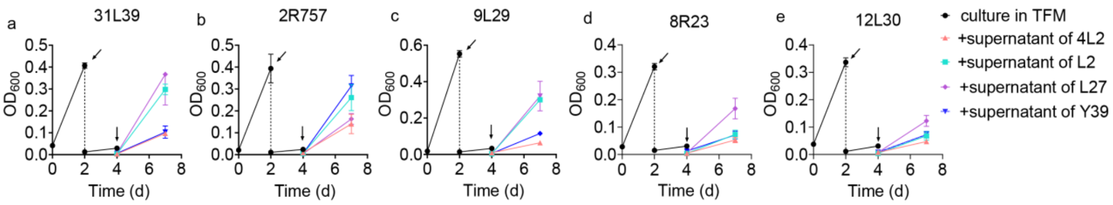

Fig. S7. Thiamine-dependence of auxotrophic on prototrophic bacteria isolated from soils. The thiamine-auxotrophic strains isolated from soils, including *Microbacterium* sp. 31L39 (a), *Massilia* sp. 2R757 (b), *Microbacterium* sp. 9L29 (c), *Massilia* sp. 8R23 (d), and *Microbacterium* sp. 12L30 are showed. All the isolates were firstly cultured in a thiamine-free medium (TFM), and dilution transfers (oblique arrows) were performed to consume the stored thiamine in the cells of the isolates. To restore growth of the isolates, during the last dilution transfer (vertical arrows), the cultures of thiamine-auxotrophic isolates were inoculated into the culture supernatant of prototrophic strain isolated from soils. Four prototrophic strains were used, including *Stenotrophomonas* sp. 4L2, *Streptomyces* sp. L2, *Pseudomonas* sp. L27, and *Sphingomonas* sp. Y39.

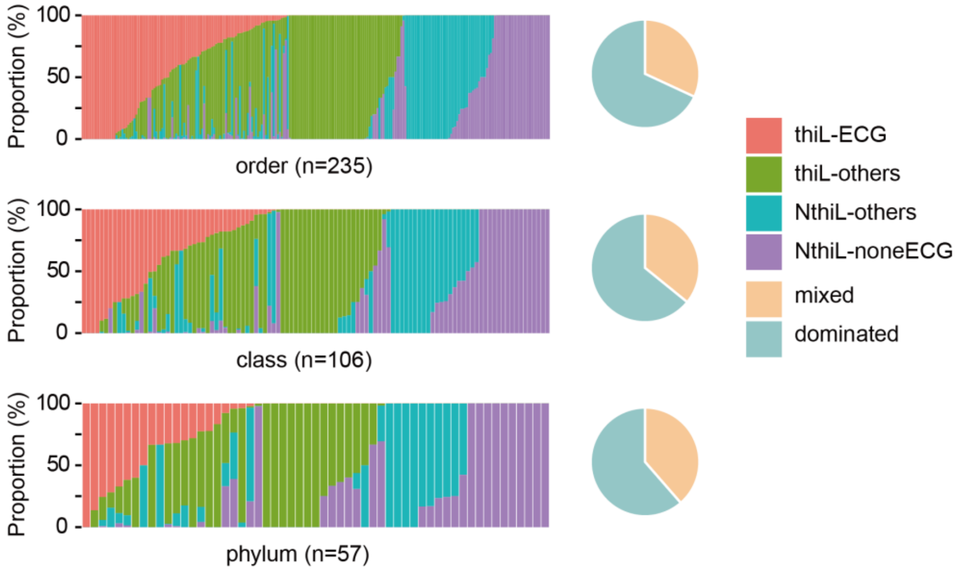

Fig. S8. Relative abundance of four types of thiamine-biosynthetic patterns at different taxonomic levels. The definitions of the four types, mixed, and dominated are same to Fig. 5.

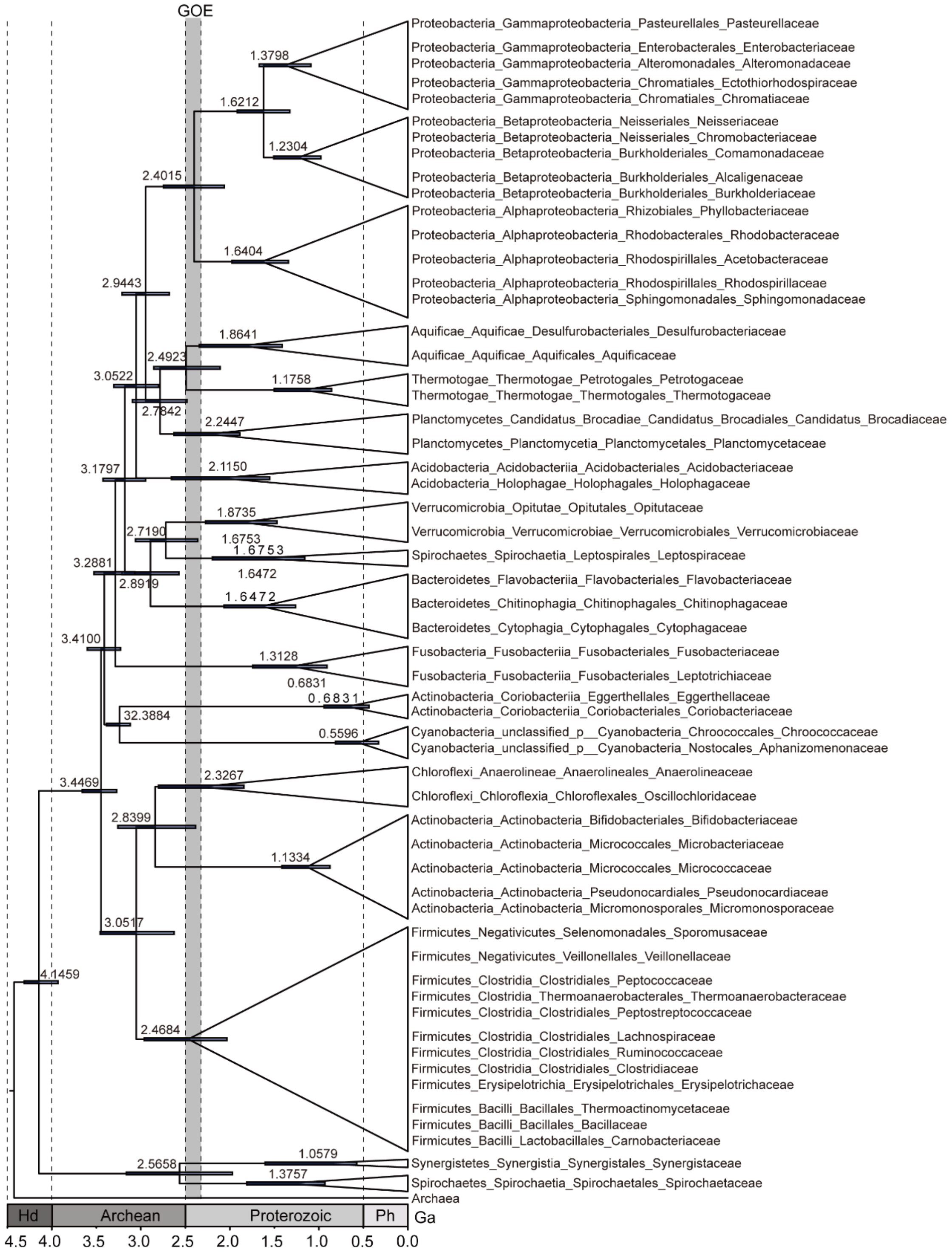

Fig. S9. Evolutionary timeline of major lineages of Bacteria. Divergent times and corresponding confidence intervals (95%) were estimated using MCMCTree based on the rooted ML concatenated tree. Timescale: Hd, Hadean; Ph, Phanerozoic; GOE, Great Oxygenation Event; Ga, billions of years.

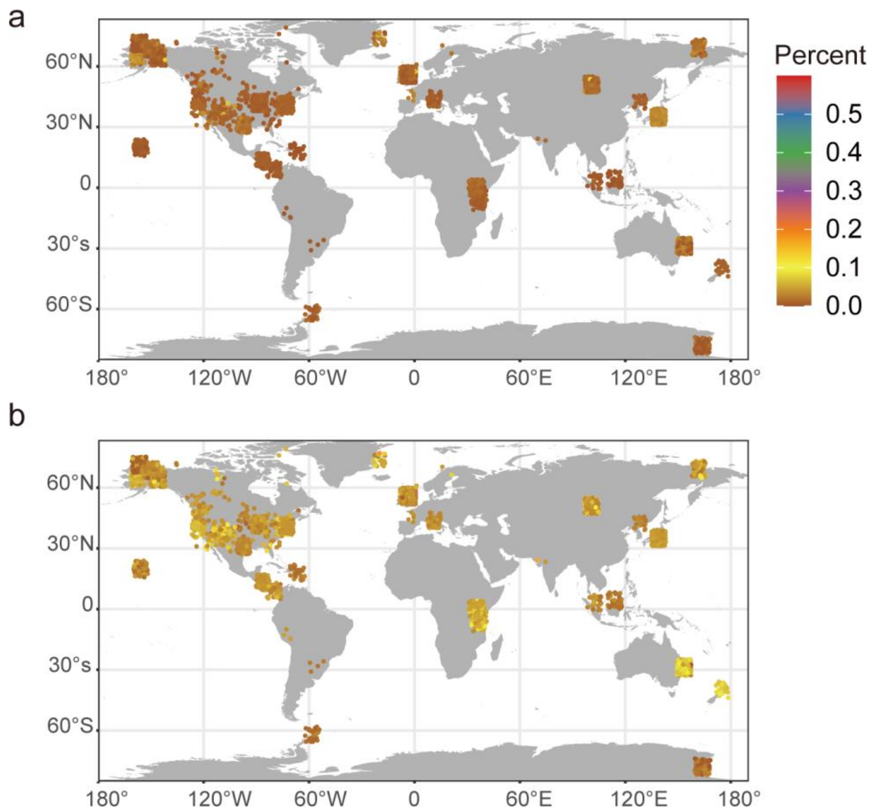

Fig. S10. Geographic and abundance distribution of thiamine-unrequired bacteria. a, NthiL-noneECG; **b**, NthiL-others. The definition of NthiL-noneECG and NthiL-others is same to Fig. 5.

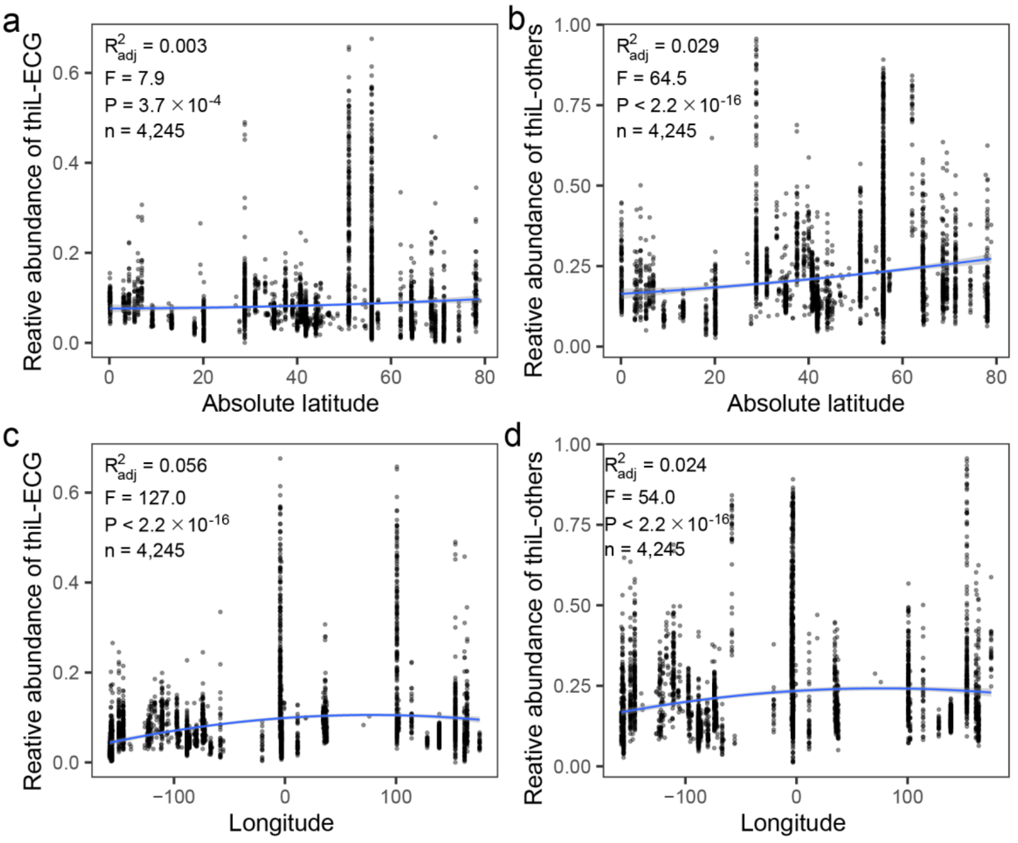

Fig. S11. The relationship between absolute latitude/longitude and the relative abundance of thiamine-prototrophic/-auxotrophic bacteria. **a**, Relationship between absolute latitude and the thiamine-prototrophic (type: thiL-ECG) bacteria. **b**, Relationship between absolute latitude and the thiamine-auxotrophic (type: thiL-others) bacteria. **c**, Relationship between longitude and the thiamine-prototrophic (type: thiL-ECG) bacteria. **d**, Relationship between absolute latitude and the thiamine-auxotrophic (type: thiL-others) bacteria. The lines represent the second-order polynomial fit based on ordinary least-squares regression, and the shaded areas represent the 95% confidence intervals.

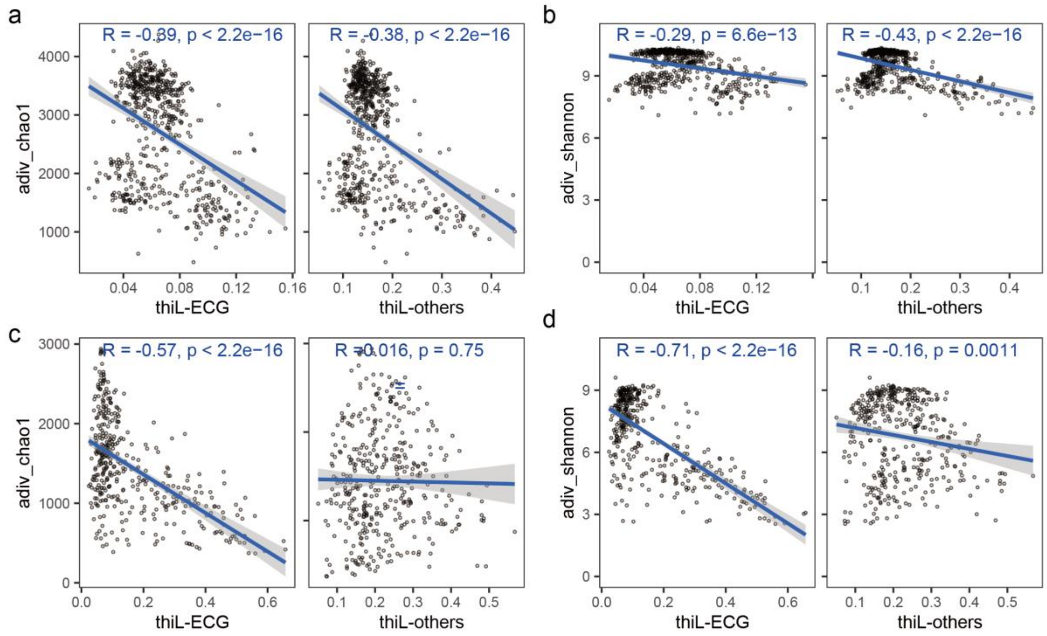

Fig. S12. The relationship between the Chao1 (a, c) or Shannon (b, d) index of the bacterial community and the relative abundance of the thiamine-auxotrophs (thiL-ECG, left) or -prototrophs (thiL-others, right). The relationships in agriculture-related (a, b) and steppe-grassland (c, d) soils are showed.
