# Supplementary material for: Bacterial growth and environmental adaptation via thiamine biosynthesis and thiamine-mediated metabolic interactions
Source: ISME J. 2024 Aug 12;18(1):wrae157. doi: 10.1093/ismejo/wrae157 (PMC11346370; doi:10.1093/ismejo/wrae157)
Supplement: SupplementaryTables_wrae157 [file supplementarytables_wrae157.pdf]

Supplementary Tables

Table S1. Primers used in this study.

Primer	Sense (5'-3')	Anti-sense (5'-3')
7D-2-*bhba*	TTACATGTCCAGCACCACCTC	ATGCGCATCTTTACCCACCG
bhba-qpcr	TGGTGGATCTTCAGCGAACC	CCCGAATACATCCGGCAGTT
gyrA-N1	TGCTCTACGCGATGTACGAC	AAGTTGCCCTGACCGTTGAT
gyrA-7D-2	ATTGCAATCCAGCAGTCCGA	TCCAGGTTGATCAGCGTCAC
thiC-7D-2	GAAGAAGACCTGCGTGTGGA	CCACATACTCCATCTCGGGC
thiE-7D-2	AAGATCTGGACGTGATCGGC	ATCTTCTTGAGCGTGGTGGG
thiG-7D-2	CAGCCCGACACACTCAATCT	CATCTGCATGGCATAGGGGT
thiP-7D-2	TGGTAGCCCTGCTGTTCAAG	ACAGGCCGCAATAAAGGACA
thiQ-7D-2	GCGCGATGTGAGTCTGGATA	ATGCACATCGGTGGTGTCTT
thiC-N1	ATCACCCAGGAGATGGCCTA	TGATCTTCACCAGGAACCGC
thiE-N1	TCTCCCACGAATTTTCGCTCT	CCTTGTCACGCACCTGGAA
thiG-N1	TGATCGGTGACGAGACCTCA	GGTGGCGTACGGAAGGAC
thiP-N1	CATCAAGTGGGCCGTCATCT	CTGCTGGTCGAAGTTGAGGT
thiQ-N1	AGTGGTGTCAACAAGCGGTA	GCGTAGTGCTGGAAGACGAA

Table S2. Strains and general features of their metabolic models constructed in the study.

Strains	Genes in model	Reactions in model	Metabolites in model
Comamonas sp. 7D-2	849	1605	1609
Pseudoxanthomonas sp. X1	717	1481	1506
Achromobacter sp. A1	1068	1650	1709
Nocardioides sp. N1	936	1641	1638

Table S3. Growth of strains cultured in MSM medium with different carbon source.

Carbon source	Strains			
	7D-2	X1	A1	N1
D-xylose	+	-	-	+
Sodium acetate	+	-	+	+

Mannitol	-	+	-	+
Trisodium citrate dihydrate	+	-	+	-
Succinic acid	+	-	+	-
D-Galacturonic acid	+	+	-	+
Glucose	+	+	+	+

Note:+ indicates that it can grow; - Indicates that it cannot grow

Table S4. Quality spectrum of standard samples.

Standard	Formula	Parent ions (m/z)	Second order fragment ions (m/z)
Sodium glutamate	C5H8NO4Na	170	56.1; 84.1
Lysine	C6H14O2N2	146.9	129; 101; 130; 119; 105
Phenylalanine	C9H11NO2	165.9	103
Uracil	C4H4N2O2	112.8	52.9; 69.9; 95.9
Proline	C5H9NO2	115.9	70.2; 43.3
Stearic acid	C18H36O2	284.5	266.7; 211.2; 183; 103.8
thiamine	C12H17ClN4OS	265.02	88.95; 121.98; 143.95

Table S5. Genomes of the four strains used in the study.

Strains	Genomes
Comamonas sp. 7D-2	GCA_022749555.1
Pseudoxanthomonas sp. X1	VCHZ01000000
Achromobacter sp. A1	GCA_008245125.1
Nocardioides sp. N1	GCA_014648255.1
