# Supplementary material for: Bacterial growth and environmental adaptation via thiamine biosynthesis and thiamine-mediated metabolic interactions
Source: ISME J. 2024 Aug 12;18(1):wrae157. doi: 10.1093/ismejo/wrae157 (PMC11346370; doi:10.1093/ismejo/wrae157)
Supplement: SupplementaryMethods_wrae157 [file supplementarymethods_wrae157.pdf]

Supplementary Methods

Strains and Culture Conditions

The strains *Pseudoxanthomonas* sp. X-1, *Comamonas* sp. 7D-2, *Nocardioides* sp. N1, and *Achromobacter* sp. A1 were isolated and collected in our lab. The strain *Escherichia coli* K-12 Δ*thiE*, a knockout mutant of *E. coli* K-12, was obtained from Horizon Discovery. All the strains were cultured on LB or mineral salt medium (MSM: 1.0 g/L of NH_4Cl, 1.5 g/L of K_2HPO_4, 0.5 g/L of KH_2PO_4, 0.2 g/L of $MgSO_4$ and 1.0 g/L of NaCl) supplemented with glucose (1.0 g/L). To isolate the thiamine-auxotrophic strains, MSM supplemented with additional nutrients (thiamine-free medium, 1 g/L of glucose, 0.01 g/L of L-proline, 0.01 g/L of L-serine, 0.01 g/L of L-tryptophan, 0.01 g/L of L-phenylalanine, 0.01 g/L of L-lysine, 0.01 g/L of L-glutamic acid, 0.01 g/L of L-arginine, 0.01 g/L of L-threonine, 0.01 g/L of L-valine, 0.01 g/L of L-methionine, 0.01 g/L of L-isoleucine, 0.01 g/L of L-histidine, 0.01 g/L of L-cysteine, 0.01 g/L of L-leucine, 0.00002 g/L of biotin, 0.00002 g/L of folic acid, 0.0001 g/L of pyridoxine HCl, 0.00005 g/L of riboflavin, 0.00005 g/L of nicotinic acid, 0.00005 g/L of pantothenic acid, 0.00005 g/L of PABA, 0.00005 g/L of cyanocobalamin, and 0.00005 g/L of thioctic acid) were used.

Assessing different cutoffs of E-values and Hit scores

Different cutoffs of E-values and Hit scores were assessed for both HMM- and BLASTP-based searches, and we chose the best Hit scores and E-values cutoff, which could partition the retrieved hits into two distinct groups, including the higher score group and the other group with a much lower score that consisted of distant homologs. Only the hits identified by both the HMM- and BLASTP-based searches were retrieved as thiamine-related genes for further analysis.

Molecular Dating of the Tree

The divergence time of the species tree was estimated with MCMCTree from PAML v4.9i [1]. The approximate likelihood calculation, independent rate model and birth–death process implemented in MCMCTree were used. Two repeated calculations were performed to observe the consistency. The clocks were calibrated with two sets of temporal constraints that were directly linked to fossil and geochemical evidence, i.e., 3.35–4.38 Bya for the age of the last universal common ancestor (root), and 3.23–4.18 Bya for Cyanobacteria [2]. Ages of fossil records were obtained from TimeTree (https://www.timetree.org/) and the literature [3,4,5].

Soil Pretreatment for Bioaugmentation

BO is a widely used, halogenated aromatic herbicide, and bromoxynil is a metabolic intermediate and active ingredient of BO. Bromoxynil (^{12}C) was obtained from Aladdin Reagent Co., Ltd. (Shanghai, China). ^{13}C-labeled bromoxynil was obtained from Cambridge Isotope Laboratories, Inc. (Tewksbury, MA). All other

chemicals and solvents were purchased from Sigma–Aldrich (St. Louis, MO, USA).

Two bacterial strains were used in the soil pretreatment experiment. *Pseudoxanthomonas* sp. X-1 could only degrade BO to bromoxynil, resulting in the accumulation of bromoxynil [6]. The strain *Comamonas* sp. 7D-2, without BO-degrading capability, could degrade bromoxynil completely [7]. The combination of X-1 and 7D-2 could mineralize BO.

Yellow cinnamon soil (0-20 cm soil layer) was collected from cropping fields located in Nanjing City, Jiangsu Province, China (N 32°01'27", E 118°51'49"). The soil was ground and passed through a 0.85-mm mesh sieve. Each 1.5 kg of soil sample was kept at 50% water-holding capacity (WHC) and placed into a plastic pot. Then, soil samples were further treated with 1) inoculation of the microbial consortium only (inoculation treatment) or 2) the combination of herbicide application and microbial consortium inoculation (inoculation-herbicide treatment). For the inoculation treatment, soils were inoculated with two strains, X-1 and 7D-2, at a ratio of 1:1, with a final concentration of approximately 2×10^8 CFU/g soil for each strain. For the inoculation-herbicide treatment, we added 5 mg/kg of BO to soil samples and inoculated both strains of X-1 and 7D-2 with the same final concentration as those used in the inoculation treatment. The BO and inoculating strains were added repeatedly every three days for a total of 10 times. Soil samples from Day 30 from each treatment were collected for further experiments. Meanwhile, the distribution of strain 7D-2 in soil samples on Day 30 was quantified using quantitative PCR (qPCR). The detailed methods for qPCR are described below. We sequenced the 16S rRNA and ITS genes of the microbiota in soils to explore the effects of bioaugmentation on the bacterial and fungal communities, respectively. The detailed methods for high-throughput sequencing are proved in Supplementary Methods.

SIP Experiment

Normal (^{12}C) and ^{13}C-labeled bromoxynil were used in the experiment. A 50 mL sterilized conical flask was filled with 20 mL of MSM and 1 g of pretreated soils, and the initial concentration of bromoxynil was set to 0.07 mM. All cultures were incubated at 30 °C and 180 rpm. Three replicates were set for each treatment. We used high-performance liquid chromatography (HPLC) to detect the residues of bromoxynil every eight hours until it was completely degraded. The HPLC process was described by Chen et al. [7].

According to the HPLC results, 80% of bromoxynil was degraded for inoculation-herbicide pretreatment, while that in inoculation pretreatment was 65% after 32 hours of processing. We selected the samples treated for 32 hours for the SIP experiment. Total DNA was extracted using the FastDNA Spin Kit (Solon, USA) according to the manufacturer's instructions. A total of 5 μg DNA was dissolved in Tris-EDTA (pH 8.0)-CsCl solution, and the buoyant density was adjusted to 1.725

g/ml. Subsequently, the solution was centrifuged at 45,000 rpm (20 ℃) for 45 hours. The buoyant density of different fractions was detected using a digital refractometer (model AR200; Leica Microsystems Inc., Buffalo Grove, IL). A total of 14 fractions were collected by a fraction recovery system (Beckman Coulter). The purity and concentration of the extracted DNA were measured using a NanoDrop spectrophotometer (ND2000, Thermo Fisher Scientific, Wilmington, DE, USA).

The purified DNA derived from fractions 5 to 12 in the DNA-SIP assay was further used to analyze the bacterial composition to identify bromoxynyl-degrading strains. Since the amount of DNA in each fraction was insufficient for sequencing, two adjacent fractions of DNA were combined into one sample. The bacterial composition was analyzed by sequencing the amplified V4-V5 regions of the bacterial 16S rRNA gene with primers 515F (5’-GTGCCAGCMGCCGCGG-3’) and 907R (5’-CCGTCAATTCMTTTRAGTTT-3’). Amplicon sequencing was performed on an Illumina MiSeq platform according to the standard protocols at Biozeron Biotechnology Co. (Shanghai, China).

Amplicon Sequencing

For soils with bioaugmentation treatments, we sequenced the 16S rRNA and ITS genes of the microbiota in soils to explore the effects of bioaugmentation on the bacterial and fungal communities, respectively. The total DNA was extracted using the E.Z.N.A.® Soil DNA Kit (Omega Bio-tek, Norcross, GA, U.S.) according to manufacturer’s protocols. The DNA quality and quantity were determined using a NanoDrop 2000 Spectrophotometer (Bio-Rad Laboratories Inc., USA.). For bacterial community analysis, the V1-V9 region of the bacteria 16S ribosomal RNA gene were amplified by PCR using the primers 27F (5’- AGRGTTYGATYMTGGCTCAG’) and 1492R (5’- RGYTACCTTGTTACGACTT -3’). For fungal community analysis, PCR amplification was performed targeting internal transcribed spacer region (ITS) using the primers ITS1F (5’- CTTGGTCATTTAGAGGAAGTAA -3’), and ITS4R (5’- TCCT CCGC TTAT TGAT ATGC -3’). Full-length 16S rRNA and ITS gene sequencing was performed on a PacBio Sequel II platform according to the standard protocols at Biozeron Biotechnology Co. (Shanghai, China).

Processing of Sequencing Data

The raw reads from PacBio system were processed to obtain a demultiplexed circular consensus sequence using SMRT Link Analysis software v.9.0. The detailed parameters used were as follows: minimum number of passes = 3, minimum predicted accuracy = 0.99. The SMRT Portal was used to filter the raw reads at lengths < 800 or >2500 bp. The obtained sequences were further filtered by removing barcode, primer sequences, chirmas and the sequences containing 10 consecutive identical bases. The operational taxonomic units (OTUs) were clustered at the 98.65% similarity level by UPARSE [8]. The raw sequences from the Illumina system were

quality-filtered by Trimmomatic [9] and merged by FLASH (10). Chimeric sequences were identified and removed using UCHIME [11]. The OTUs were analyzed using UPARSE, and sequences were assigned to OTUs at a 97% similarity cutoff. The RDP classifier [12] was used for taxonomic annotation of each OTU at the 70% threshold. The α- and β-diversity were analyzed using the QIIME pipeline [13]. Principal coordinates analysis (PCoA) based on Bray–Curtis similarities was performed using the "vegan" function in R v4.1.1. The significant difference in community structure composition between the ^{12}C and ^{13}C samples was analyzed by the Kruskal–Wallis H test in R v4.1.1.

Reconstruction of Single-Species Models

A total of four strains were selected for GSMM construction. In addition to strains X-1 and 7D-2, we also chose strains N1 and A1, which were regarded as keystone strains revealed by SIP analysis. Genomes of the four strains were obtained from public resources (Table S5). The draft metabolic models of each strain were reconstructed separately by analyzing the annotated genome sequence through ModelSEED [14]. We tested the growth of each strain in MSM with different carbon sources (Fig. S4, Table S3), and these experimental results were used for model curation. The draft model was curated in MATLAB using COBRAToolbox-3.0 [15]. If the draft model failed to produce biomass at a specific carbon source where the growth of the strain was feasible, as demonstrated by the experimental results, potential missing reactions were identified and added to the draft model. These potential missing reactions were identified using the FastGapFill function, and only reactions with gene evidence in the genomes of the strain or species closely related to them by phylogeny were preserved. Missing reactions were further artificially supplemented according to public databases, such as KEGG [16], UniProt [17], BiGG [18], IMG [19], and MetaCyc [20]. Invalid or incorrect energy-generating loops were removed. Element disequilibrium reactions based on chemical formulae were checked and balanced. After iterative calibration, the reconstructed model could generate all biomass components in MSM with alternative carbon sources, which was consistent with the experimental results.

Biomass of the community model

The biomass of the consortium was set as the sum of the biomass of each strain. According to the amplicon sequencing results of community composition in soils, the relative abundance of strain 7D-2 in the community was higher than 80%. Therefore, the biomass ratio of 7D-2 and other strains in combinations was set to 9:1 (two-strain community), 8:1:1 (three-strain community), and 7:1:1:1 (four-strain community) for simulation.

Testing the Computational Predictions

To test the computational predictions of bromoxynil-degrading efficiencies by

different consortia experimentally, bromoxynil-degrading rates by strain 7D-2 and/or different strain combinations in both MSM and *in situ* soils were measured. The setting of strain combinations and proportions were consistent with those in community model construction. For the flask experiment, strains in each combination (OD_{600} = 0.3 for each strain) were inoculated into a 50 mL sterilized conical flask containing 20 mL MSM with 0.07 mM bromoxynil and enriched at 30 ℃ and 180 rpm for 21 hours. Three replicates were set for each treatment. The residue of bromoxynil was detected every 6 hours by HPLC using the methods described above. For the *in situ* soil experiment, the soil was mixed with 10 mg/kg of BO. Strains in each combination were inoculated into the corresponding soils with a final concentration of approximately 2×10^6 CFU/g soil for each combination. Soil samples were placed in a light incubator with 25/22 ℃ day/night temperatures. Three replicates were set for each treatment. After treatment for seven consecutive days, 10 g of soil was collected for determination of BO residue by HPLC. The abundance of the inoculated strain 7D-2 was also detected at 0, 2, 3, and 5 days by qPCR.

For qPCR, 0.25 g soil samples were used for DNA extraction using the FastDNA Spin Kit (Solon, USA) according to the manufacturer's instructions. Both the nitrilase gene Bxn2 and the specific 16S rRNA sequence of strain 7D-2 were chosen to represent the population of strain 7D-2. The Bxn2 and 16S rRNA sequences were amplified, and the PCR product was cloned and inserted into a pMDTM19-T vector. The standard curves were constructed with 10-fold serial dilutions of pMDTM19-T. The corresponding copy numbers of the Bxn2 and 16S rRNA genes were calculated using the method of Lee et al. [21]. The threshold cycle (Ct) values in each dilution were measured in duplicate using qPCR, which was performed on an ABI StepOnePlus real-time PCR system with a TB Green Premix Ex Taq (Tli RNaseH Plus) kit (Takara Bio). Absolute copy numbers of target genes were calculated using the Ct values based on the standard curves. All qPCR analyses were carried out in triplicate for each sample.

The predicted metabolic interactions among strains were experimentally tested by detecting secreted metabolites in cocultures and supporting the growth of strains by the secreted metabolites. The consortium (7D-2+N1+A1) was used to detect secreted metabolites by different strains. The strains 7D-2, N1 and A1 (OD_{600} = 0.3 for each strain) were cocultured in nitrogen-free MSM containing 0.2 mM bromoxynil as the sole carbon and nitrogen source at 30 ℃ for 6 h. Secretions by 7D-2, the N1, and A1 in cocultures were collected and screened by LC–MS. The LC–MS process was described by Ruan et al. [22].

To verify the predictions that strain 7D-2 could use thiamine provided by other strains, we tested whether extra thiamine could improve the growth of strain 7D-2. To this end, strain 7D-2 was cultured in MSM with 0.07 mM bromoxynil and different

concentrations of thiamine (0, 1, 3, 5, 7, 10, 50, 100 μM), and the biomass of strain 7D-2 was detected. Similarly, the supporting growth of strains by the secreted metabolites provided by other strains was tested by comparing the growth of strains cultured in medium supplemented with corresponding secreted metabolites vs. those without supplementary secreted metabolites.

Strain Isolation and Analysis of BO/Bromoxynil-Degrading Ability.

To obtain the keystone strains for BO/bromoxynil degradation, soil samples from the inoculation-herbicide treatment were used for isolation of strains. The strains were isolated from soils by dilution separation methods on LB agar, and the 16S rRNA gene was amplified using primers 27F (5'-AGAGTTTGATCMTGGCTCAG-3') and 1492R (5'-ACGGYTACCTTGTTACGACTT-3'). The taxonomic position of the isolates was analyzed using the 16S rRNA gene sequence obtained by the EzTaxon server [23]. A phylogenetic tree of isolates was constructed using the neighbor-joining (NJ) method (bootstraps = 1,000) in MEGA11 [24]. The ability of isolates to degrade BO/bromoxynil was explored. For the degradation of bromoxynil, the cells of the strain were inoculated at 2% volume into 20 mL of MSM containing 0.18 mM bromoxynil. All cultures were incubated at 30 ℃ and 180 rpm for 16 h. Bromoxynil was detected by HPLC using the methods described by Chen et al. [7]. For the degradation of BO, BO insoluble in a water-forming emulsion was used for detection. Single colonies were selected on LB agar plates with 1.4 mM BO. After the plate was inverted at 30 ℃ for 24 h, hydrolytic circles around the colonies were observed. The main operational taxonomic units (OTUs) with significant differences between ^{12}C and ^{13}C samples that were also isolated from soils were identified as specific keystones for further model construction.

Isolation of Thiamine-Auxotrophic Strains

The isolates obtained from soils were cultured in thiamine-free medium for 48 h, and then transferred into new thiamine-free medium (1% v/v) every 48 h for 2-5 times to consume the stored thiamine in their cells. Then the exogenous thiamine (20 μL, 0.05 g/L) was added to the thiamine-free medium (20 mL) to restore growth of the isolates. Thiamine-auxotrophic isolates were identified if they were unable to grow after depleting all stored thiamine, but resumed growth after the addition of exogenous thiamine. *E. coli* and its thiamine-auxotrophic mutant Δ*thiE* were used as controls. To restore the growth of thiamine-auxotrophic strains by thiamine-prototrophic strains, the culture supernatant of *E. coli* in the thiamine-free medium was collected by centrifugation at 12,000 rpm for 5 min and passed through a 0.22 μm sterile filter to remove remaining cells. The culture of thiamine-auxotrophic strain (200 μL) was added to the culture supernatant of *E. coli* (20 mL) to restore its growth. The culture supernatant of *E. coli* K-12 Δ*thiE* was used as a control. All the strains were cultured at 30 ℃, and the growth of each strain was determined by

measuring the OD_{600}.

References

1. Yang Z. PAML 4: Phylogenetic Analysis by Maximum Likelihood. *Mol Biol Evol*. 2007;24:1586–1591.
2. Dos Reis M, Donoghue PCJ, Yang Z. Bayesian molecular clock dating of species divergences in the genomics era. *Nat Rev Genet*. 2016;17:71–80.
3. Chen S, Sun G, Yan Y, Konstantinidis KT, Zhang S, Deng Y, *et al*. The Great Oxidation Event expanded the genetic repertoire of arsenic metabolism and cycling. *Proc Natl Acad Sci USA*. 2020;117:10414–10421.
4. Betts HC, Puttick MN, Clark JW, Williams TA, Donoghue PCJ, Pisani D. Integrated genomic and fossil evidence illuminates life's early evolution and eukaryote origin. *Nat Ecol Evol*. 2018;2:1556–1562.
5. Satkoski AM, Beukes NJ, Li W, Beard BL, Johnson CM. A redox-stratified ocean 3.2 billion years ago. *Earth Planet Sc Lett*. 2015;430:43–53.
6. Ruan Z, Cao W, Zhu J, Yang B, Jiang J, Chen C, *et al*. Comparative Genomic Analysis of Pseudoxanthomonas sp. X-1, a Bromoxynil Octanoate-Degrading Bacterium, and Its Related Type Strains. *Curr Microbiol*. 2022;79:65.
7. Chen K, Liu Y, Mao D, Liu X, Li S, Jiang J. An Essential Esterase (BroH) for the Mineralization of Bromoxynil Octanoate by a Natural Consortium of Sphingopyxis sp. Strain OB-3 and Comamonas sp. Strain 7D-2. *J Agr Food Chem*. 2013;61:11550–11559.
8. Edgar RC. UPARSE: highly accurate OTU sequences from microbial amplicon reads. *Nat Methods*. 2013;10:996–998.
9. Bolger AM, Lohse M, Usadel B. Trimmomatic: a flexible trimmer for Illumina sequence data. *Bioinformatics*. 2014;30:2114–2120.
10. Magoč T, Salzberg SL. FLASH: fast length adjustment of short reads to improve genome assemblies. *Bioinformatics*. 2011;27:2957–2963.
11. Edgar RC, Haas BJ, Clemente JC, Quince C, Knight R. UCHIME improves sensitivity and speed of chimera detection. *Bioinformatics*. 2011;27:2194–2200.
12. Caporaso JG, Lauber CL, Walters WA, Berg-Lyons D, Lozupone CA, Turnbaugh PJ, *et al*. Global patterns of 16S rRNA diversity at a depth of millions of sequences per sample. *Proc Natl Acad Sci USA*. 2011;108:4516–4522.
13. Caporaso JG, Kuczynski J, Stombaugh J, Bittinger K, Bushman FD, Costello EK, *et al*. QIIME allows analysis of high-throughput community sequencing data. *Nat Methods*. 2010;7:335–336.
14. Seaver SMD, Liu F, Zhang Q, Jeffryes J, Faria JP, Edirisinghe JN, *et al*. The ModelSEED Biochemistry Database for the integration of metabolic annotations and the reconstruction, comparison and analysis of metabolic models for plants, fungi and microbes. *Nucleic Acids Res*. 2021;49:D575–D588.
15. Heirendt L, Arreckx S, Pfau T, Mendoza SN, Richelle A, Heinken A, *et al*. Creation and analysis of biochemical constraint-based models using the COBRA Toolbox v.3.0. *Nat Protoc*. 2019;14:639–702.
16. Kanehisa M, Sato Y, Morishima K. BlastKOALA and GhostKOALA: KEGG Tools for Functional Characterization of Genome and Metagenome Sequences. *J Mol Biol*. 2016;428:726–731.

17. UniProt Consortium. UniProt: a worldwide hub of protein knowledge. *Nucleic Acids Res*. 2019;47:D506–D515.
18. Norsigian CJ, Pusarla N, McConn JL, Yurkovich JT, Dräger A, Palsson BO, *et al*. BiGG Models 2020: multi-strain genome-scale models and expansion across the phylogenetic tree. *Nucleic Acids Res*. 2020;
19. Chen IA, Chu K, Palaniappan K, Pillay M, Ratner A, Huang J, *et al*. IMG/M v.5.0: an integrated data management and comparative analysis system for microbial genomes and microbiomes. *Nucleic Acids Res*. 2019;47:D666–D677.
20. Caspi R, Billington R, Keseler IM, Kothari A, Krummenacker M, Midford PE, *et al*. The MetaCyc database of metabolic pathways and enzymes-a 2019 update. *Nucleic Acids Res*. 2020;48:D445–D453.
21. Lee C, Kim J, Shin SG, Hwang S. Absolute and relative QPCR quantification of plasmid copy number in Escherichia coli. *J Biotechnol*. 2006;123:273–280.
22. Ruan Z, Xu M, Xing Y, Jiang Q, Yang B, Jiang J, *et al*. Interspecies Metabolic Interactions in a Synergistic Consortium Drive Efficient Degradation of the Herbicide Bromoxynil Octanoate. *J Agr Food Chem*. 2022;70:11613–11622.
23. Chun J, Lee J, Jung Y, Kim M, Kim S, Kim BK, *et al*. EzTaxon: a web-based tool for the identification of prokaryotes based on 16S ribosomal RNA gene sequences. *Int J Syst Evol Micr*. 2007;57:2259–2261.
24. Saitou N, Nei M. The neighbor-joining method: a new method for reconstructing phylogenetic trees. *Mol Biol Evol*. 1987;4:406–425.
